# Active learning of alchemical adsorption simulations; towards a universal adsorption model†

**DOI:** 10.1039/d4sc02156h

**Published:** 2024-10-08

**Authors:** Etinosa Osaro, Fernando Fajardo-Rojas, Gregory M. Cooper, Diego Gómez-Gualdrón, Yamil J. Colón

**Affiliations:** a Department of Chemical and Biomolecular Engineering, University of Notre Dame IN 46556 USA ycolon@nd.edu; b Department of Chemical and Biological Engineering, Colorado School of Mines 1500 Illinois St Golden CO 80401 USA

## Abstract

Adsorption is a fundamental process studied in materials science and engineering because it plays a critical role in various applications, including gas storage and separation. Understanding and predicting gas adsorption within porous materials demands comprehensive computational simulations that are often resource intensive, limiting the identification of promising materials. Active learning (AL) methods offer an effective strategy to reduce the computational burden by selectively acquiring critical data for model training. Metal–organic frameworks (MOFs) exhibit immense potential across various adsorption applications due to their porous structure and their modular nature, leading to diverse pore sizes and chemistry that serve as an ideal platform to develop adsorption models. Here, we demonstrate the efficacy of AL in predicting gas adsorption within MOFs using “alchemical” molecules and their interactions as surrogates for real molecules. We first applied AL separately to each MOF, reducing the training dataset size by 57.5% while retaining predictive accuracy. Subsequently, we amalgamated the refined datasets across 1800 MOFs to train a multilayer perceptron (MLP) model, successfully predicting adsorption of real molecules. Furthermore, by integrating MOF features into the AL framework using principal component analysis (PCA), we navigated MOF space effectively, achieving high predictive accuracy with only a subset of MOFs. Our results highlight AL's efficiency in reducing dataset size, enhancing model performance, and offering insights into adsorption phenomenon in large datasets of MOFs. This study underscores AL's crucial role in advancing computational material science and developing more accurate and less data intensive models for gas adsorption in porous materials.

## Abstract 2

Active learning facilitated adsorption predictions in porous materials by incorporating “alchemical” molecules, fugacity, and material features (PC1, PC2) using Gaussian process regression.

## Graphical abstract

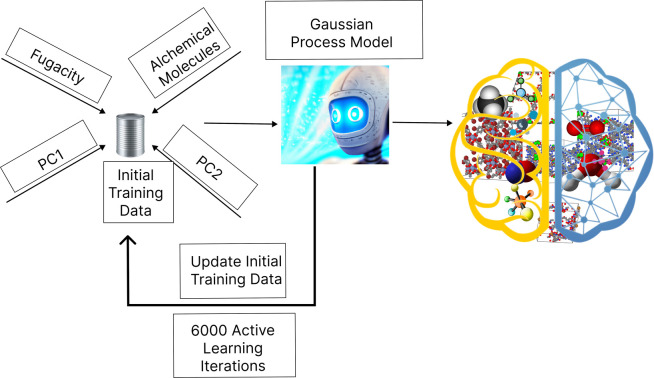

## Introduction

Metal–organic frameworks (MOFs) stand as versatile porous materials with exquisitely tunable structures, and tremendous potential for numerous applications across various fields.^1–6^ A large fraction of these applications seek to exploit the adsorption properties of these materials, which are composed of interconnected building blocks (*i.e.*, metallic nodes and organic linkers).^7–10^ For instance, numerous MOFs could be imparted with adsorption properties to substitute *ca.* 80% of heat-based chemical separations processes with adsorption-based ones.^11^ Therefore, harnessing the full potential of MOFs, and accelerating their development by anticipating MOF designs that embody desired properties through computation requires reliable predictions of adsorption behavior within these intricate frameworks.

The vast MOF space, spanning countless unique structures formed from different combinations of constitutive building blocks, poses an immense challenge to predicting gas adsorption behaviors across this expansive space of materials sufficiently fast. Depending on the complexity of the molecule model, predicting the adsorption loading of a molecule within a MOF through classical techniques such as grand canonical Monte Carlo (GCMC) simulations^12–16^ may require substantial and specialized computational resources. Each GCMC simulation involves comprehensive exploration of the configurational space of gas molecules within MOF pores, calculating interaction energies, and sampling numerous adsorption states. Regardless of the complexity of the molecule model, the computational expense escalates significantly as the number of MOFs, adsorbate molecules, and adsorption conditions under consideration increase.

Machine learning (ML) seems poised to be an important tool to predict adsorption in MOFs.^17–22^ However, developing ML that can comprehensively navigate the immense space formed by different MOF and molecule pairings demands a high volume of training data to achieve reliable predictions. Acquiring such large datasets can be an arduous, time-consuming, and computationally expensive task. Several ML adsorption models documented in the literature demand an extensive dataset ranging from thousands to millions of data points. Our solution, active learning (AL), circumvents this necessity.

To circumvent the above issue, AL could be used as a strategic approach to optimize the data acquisition process. AL, a subfield of ML, reduces the data burden to train a model through an iterative effort that guides the collection of training data only towards the most informative data points, while simultaneously using these data points to train a surrogate model to predict the quantity of interest and the uncertainty associated with the prediction.^23–27^ In this work, we will add data to the training using the points for which the prediction uncertainty is highest.

We envision AL to play a crucial role in the development of ML adsorption models by guiding the selection of adsorption scenarios that offer surrogate models the most information about adsorption behavior, thereby reducing the computational expense associated with conducting GCMC simulations to generate training data. We select specific combinations of MOFs, molecules, and conditions that contribute to the surrogate model's predictions of adsorption.

AL has been demonstrated to reduce the data burden to train models that predict adsorption of specific molecules. For instance, in a previous study, Osaro and coworkers^28^ demonstrated the development of a model to predict full pure gas isotherms for methane, nitrogen, hydrogen and carbon dioxide using few training datapoints across eleven MOFs. In another instance, Mukherjee and coworkers^29^ used AL to develop a model to predict full isotherms for methane and carbon dioxide in HKUST-1 at different temperatures. AL has additionally been employed to train a model capable of predicting adsorption behaviors for various gas pairs, including xenon–krypton, carbon dioxide–methane, and hydrogen sulfide–carbon dioxide, within a single MOF.^30^

The ultimate goal of this work is to facilitate the development of adsorption models of any gas in any porous material. In this paper, we make progress towards that goal by reducing the data burden of alchemical adsorption in MOFs.

Training of a universal adsorption model implies presenting the model with adsorption data for different molecules, along with some representation of said molecules. As molecules can be modeled using some combinations of values for parameters in intermolecular (*e.g.*, Lennard-Jones and coulombic parameters) and intramolecular potentials, Gómez-Gualdrón and coworkers showed that to “teach” a model about adsorption, one does not need to limit the adsorption data to real molecules. Specifically, they created 200 alchemical molecules using arbitrary combinations of potential parameters,^31^ obtained adsorption data for them using molecular simulation and used the data to train a multi-layer perceptron (MLP) model capable of predicting full isotherms for real, small, non-polar, near-spherical, rigid molecules.

The above MLP demonstrates the concept of a ML-based universal adsorption model to be sound. Yet, the feasibility of a truly universal model is contingent on the ability to incorporate sufficient adsorption data for molecules (real or alchemical) with more diverse sizes, shapes, polarity, and flexibility. However, the above MLP required approximately 5 million GCMC data points, encompassing adsorption data for 200 small, non-polar, near-spherical, rigid alchemical adsorbates, on a relatively small set of 1800 topologically and chemically diverse ToBaCCo^32^-generated MOFs, at fugacities of 0.01, 0.025, 0.05, 0.075, 0.1, 0.25, 0.5, 0.75, 1, 5, 10, 50, 75, and 100 bar.^31^ Overall, each MOF required about 2800 adsorption data points for training.

In the work above, alchemical adsorbates were adequately represented by four features, which were explored in a somewhat exhaustive fashion. Expansion of the training data to include molecules with more diverse sizes, shapes, polarity, and flexibility would require representing the adsorbates with more features, increasing the dimensionality of the adsorbate space, whose exhaustive exploration would imply an intractable number of GCMC simulations. Thus, a critical bottleneck that needs to be overcome to truly open the path towards a universal adsorption model is to gain the ability to efficiently explore the adsorbate (plus adsorbent) space.

Crucially, in this work we demonstrate for the first time the ability of AL to cut down the size of training datasets that includes adsorption data of multiple molecules, using the adsorbate space explored earlier by Gómez-Gualdrón and coworkers as a testbed. We first approached this task on a per MOF strategy by using AL to generate the training data for each MOF (1800 MOFs in total), which resulted in 57.5% data savings. The resulting surrogate models from AL per MOF are used to generate training data for a new MLP model, which was shown to retain the original predictive performance of the original MLP by Gómez-Gualdrón and coworkers. Encouraged by the results, we then approach this AL task on a joint MOF-adsorbate basis (alchemical adsorbates and 3445 MOFs). Excitingly, this approach results in drastic data savings of 99.8%. Lastly, we analyze the AL process, focusing on its selected features as the model is developed, providing insights into AL campaigns for adsorption.

## Methods

Gaussian process regression (GPR) is a probabilistic ML technique effectively used for non-linear regression tasks. It operates on the principles of Bayesian statistics and assumes a prior distribution over functions, defining a distribution over the entire space of functions that could describe the underlying data.

The fundamental concept behind GPR involves modeling the relationship between input features (predictors) and output variables (predictions) using Gaussian processes (GPs). GPs are defined by a mean function and a covariance function (also known as kernel function). The mean function represents the average trend of the data, while the covariance function captures the similarity between pairs of data points *x* and *x*′. The GPR is mathematically represented by *f* ∼ GP(*m*(*x*), *K*(*x*, *x*′)), where the function *f* has a GP distribution with mean function (*m*) and covariance function (*K*). Here, as *K* we use the rational quadratic (RQ) kernel, which takes the mathematical form:
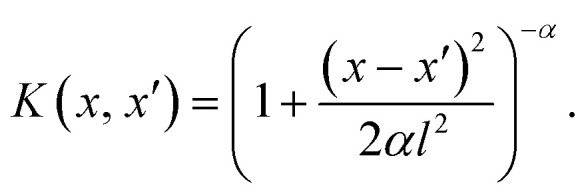


The kernel is characterized by *x* − *x*′ which is the Euclidean distance between *x* and *x*′ data points (the input data); *l* is a parameter that signifies the length scale, defining the characteristic length over which variations in the function occur, and *α* plays a pivotal role governing the balance between large-scale and small-scale fluctuations within the function.

In this study of gas adsorption in MOFs, GPR was utilized to model the relationship between various features associated with MOFs and the adsorption of specific molecules. On the navigation of fugacity and alchemical molecules across 1800 MOFs, the applied GP model trained individual GPR models for each MOF to predict the adsorption behavior of different molecules within that MOF. In the scenario involving the navigation of fugacity, alchemical molecules, and the 3445 MOFs represented by the principal component derived from their textural properties, a single GP model is trained to predict adsorption. The complete training process was carried out through AL iterations strategically selecting data points that improve the predictive accuracy of the model, ultimately reducing computational cost to generate the training data. The GPflow library^33^ was used for implementing the AL workflow and the GP used the rational quadratic (RQ) kernel.^34–37^

### AL algorithm implementation on fugacity and adsorbates

**Fig. 1:**
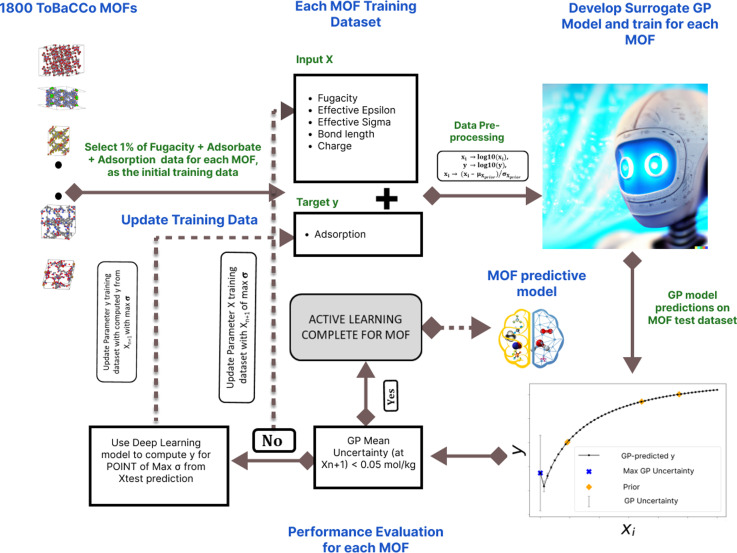
AL framework on alchemical molecules. The GPR model with the input features of fugacity, effective epsilon, effective sigma, bond length, and charge is used to predict the target variable of adsorption loading, after the model has been trained with some initial training data. The test data set (the test input features) with the highest predicted GP uncertainty is fed into the MLP model and the ground-truth is computed. This point is added to the initial training data and the model is retrained and the predictions on the test dataset is done again. This is done iteratively until the GP mean uncertainty is less than 0.05 mol kg^−1^. This procedure is conducted independently in each of the 1800 MOFs. Prior as used in this figure is the initial training data.

The study used an AL algorithm to navigate adsorption scenarios on 1800 MOFs. The features of the GP model (*F*) used for each MOF are fugacity (*f*), surrogate Lennard-Jones parameters epsilon effective (*ε*) and sigma effective (*σ*), bond length (*l*), and charges (*q*); these are the parameters that the AL algorithm automatically selects at each iteration. The GP model uses those features to make adsorption predictions in the MOF (*N*), mathematically *N* ∼ *F*(*f*, *ε*, *σ*, *l*, *q*). Fig. 1 illustrates the AL convergence criteria set at 0.05 mol kg^−1^ GP mean uncertainty and the algorithm workflow across iterations for all MOFs analyzing GP mean uncertainty and *R*^2^ behavior.

The choice of a GP mean uncertainty threshold of 0.05 mol kg^−1^ was based on empirical validation to balance predictive accuracy and computational efficiency. There is no universally accepted standard for GP uncertainty thresholds in AL, particularly in the context of adsorption modeling in porous materials. The fixed value of 0.05 mol kg^−1^ was selected to provide a consistent stopping criterion across diverse MOFs and adsorbates, ensuring robust model performance without being overly sensitive to variations in adsorption magnitudes.

Preliminary experiments where we compared two AL policies demonstrated that this threshold consistently maintained high predictive accuracy across a broad set of MOFs while reducing the number of required data points, aligning with the study's objective of minimizing computational load. These results can be seen in the section “Active Learning policy choice evaluation” in the ESI.† To summarize those results, the GP mean uncertainty policy of 0.05 mol kg^−1^ outperforms the GP maximum relative error of 2% policy in both predictive accuracy and data savings. While alternative thresholds, including ratio or percentage-based criteria, could be further explored in future research, the chosen threshold was found to be optimal for the current study's goals.

### Multilayer perceptron (MLP) model training

A unified training data set comprising approximately 2.1 million data points from multiple MOFs was created using the AL process. A Multilayer Perceptron (MLP) model was trained using TensorFlow.^38^ The selected model configuration included 500 epochs a batch size of 128, and a learning rate of 0.00001. The MLP architecture featured an input layer followed by three hidden layers, each employing Leaky ReLU^39^ activation functions.

### AL algorithm implementation on fugacity, adsorbates, and adsorbents

Principal components (PCs) of the MOF textural properties were generated using scikit-learn.^40,41^ This was applied to a dataset containing 3445 MOFs textural properties to identify primary dimensions significantly contributing to variance. The AL process was identical as before but adding PC1 and PC2 as input features to the AL process: *N* ∼ *F*(*f*, *ε*, *σ*, *l*, *q*, PC1, PC2).

### Bagging approach for model testing on fugacity, adsorbates, and adsorbents section

The GCMC simulation data, exceeding 5 million points and taken from previous studies^31,42^ was segmented into 100 bags to represent diverse adsorbates across 3445 MOFs. Ensuring that each bag encompassed the PCs of all MOFs for uniformity and representativity in each bag. These bags were structured to vary across fugacity and adsorbate types.

## Results and discussions

### AL on alchemical molecules

The MLP previously developed by Gómez-Gualdrón and coworkers^31^ used approximately 5 million training data points derived from GCMC simulations involving 200 alchemical adsorbates across 1800 MOFs. It established strong correlations between the predictions generated by the MLP model and the adsorption results obtained from GCMC simulations and will be used as a surrogate for GCMC in this study section. In this section, we demonstrate the efficacy of AL in developing a similarly predictive model, while reducing the training dataset on a per MOF basis. We constructed a new MLP model capable of predicting the adsorption behavior of real molecules using training data originated from the surrogate GP models developed by AL for each MOF. Namely, with our first AL approach, we executed the AL process in each MOF separately, and subsequently amalgamated the training datapoints selected by AL for each of all 1800 MOFs into a unified dataset. The latter was then utilized to train a new MLP model, which was tested to predict adsorption of real molecules.

The AL algorithm for the above approach is illustrated in Fig. 1. The adsorbate features that the Gaussian Process (GP) model (*F*) developed for each MOF uses to make adsorption predictions (adsorption loading *N*) are adsorbate surrogate Lennard-Jones parameters (effective epsilon (*ε*) and effective sigma (*σ*)), bond length (*l*), and charge (*q*). The adsorbate (which is defined by the combination of values of the aforementioned parameters) and fugacity (*f*) combinations to be iteratively added to the training data are automatically selected by the AL. To commence the AL algorithm for each MOF, we curated an initial set of twenty-six training data points encompassing fugacities, diverse alchemical adsorbates, and their respective adsorption values. These data points were chosen to represent a broad range derived from the training dataset. A sample of the initial training data for a single MOF is available in the project's GitHub repository.

The logarithms of the adsorbate features were used as input to the GP model, except in the case of charge and bond-length. All features underwent *z*-score standardization before being inputted into the GP model, which utilized a rational quadratic (RQ) kernel to perform the regression. At each AL iteration, the GP model trained (using the initial training dataset) for each MOF was used to compute the GP mean uncertainty for each prediction, which is a direct output of the GP model.

Following training, the model was utilized to predict adsorption based on randomly chosen values of alchemical parameters and fugacities, referred to as testing data, as detailed in Table S1 of the ESI.† Importantly, all features in the testing dataset fell within the bounds of the parameters of the training data.

The point in the testing dataset with the maximum GP uncertainty was identified and fed into the Gómez-Gualdrón and coworkers MLP model to compute the considered ground truth adsorption value, as it earlier proved to have accurate correlations with adsorption from GCMC simulations.^31^ Adsorbate and fugacity combinations continued to be added iteratively to the training data until the mean predicted uncertainty of the GP was under 0.05 mol kg^−1^. Once the threshold was met, the final GP model was utilized to predict the adsorption in the testing dataset. For each MOF, the entire testing dataset is inputted into the MLP model to generate the MLP ground truth adsorption values for comparisons with the GP predicted adsorption. We can use the MLP model to generate the ground truth data because of the high accuracy of the model when compared to GCMC simulations.^31^

**Fig. 2:**
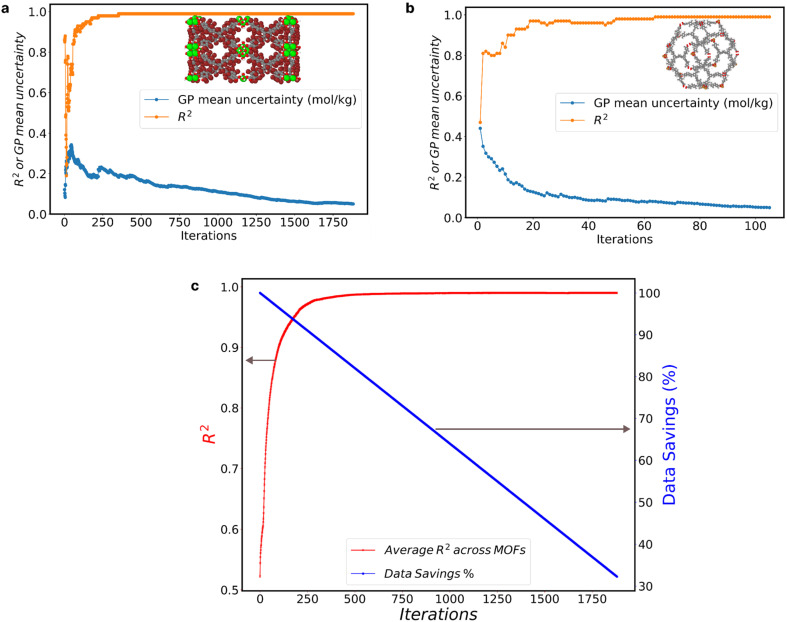
Perfomance of GPR model across MOFs and data savings. (a) Evolution of the GP mean uncertainty and *R*^2^ of the MOF with the most AL iterations; (b) evolution of the GP mean uncertainty and *R*^2^ of the MOF with the fewest AL iterations; (c) average *R*^2^ across all MOFs (left *y*-axis) and the corresponding % data savings (right *y*-axis) at various AL iterations.

We assessed the GP model performance by calculating the *R*^2^ between the “MLP ground truth” and the GP model predictions. Fig. 2 presents the evolution of *R*^2^ and GP model uncertainty for the two most extreme cases within the 1800 MOFs. The one that required the most AL iterations to reach the 0.05 mol kg^−1^ (Fig. 2a), and the one that required the fewest iterations (Fig. 2b).

The MOF with the highest number of AL iterations of 1882, initially had a GP mean uncertainty of 0.142 mol kg^−1^ and an *R*^2^ value of 0.68 in the first iteration. Over subsequent iterations, it achieved a final GP mean uncertainty of 0.049 mol kg^−1^, accompanied by an *R*^2^ value of 0.99. Conversely, the MOF with the fewest AL iterations, only 106, started with a higher initial GP mean uncertainty of 0.496 mol kg^−1^ and a lower *R*^2^ value of 0.46. However, it also reached a final *R*^2^ value of 0.99 at a GP mean uncertainty of 0.049 mol kg^−1^. Notably, fluctuations in the GP mean uncertainty (represented by the blue line) closely corresponded to fluctuations in the *R*^2^ value (represented by the orange line). These fluctuations highlight the correlation between GP mean uncertainty and *R*^2^ values, emphasizing the impact of iterative data inclusion on model performance.

The two cases above show that the GP model can predict the adsorption of adsorbates with the prescribed ending threshold regardless of the starting quality of the GP model. Albeit the efficacy (*i.e.*, number of iterations) with which AL achieves the desired goal clearly differs across MOFs.

In Fig. S3a,† we illustrate how the textural properties of MOFs (largest pore diameter (LPD), pore limiting diameter (PLD), void fraction (VF), surface area (SA), pore size standard deviation (PSSD), and inverse framework density (IFD)) influence the number of AL iterations. Our findings indicate that MOFs with low SA and IFD tend to require more iterations. Additionally, in Fig. S3b,† we represent the structural features using principal components and observe that MOFs requiring more than 1000 AL iterations predominantly cluster within a specific region in the principal component space, suggesting a similarity in their structural features. Further analysis is done in the ESI.†

The percentage of data savings, as a function of AL iterations can be calculated by eqn (1):1
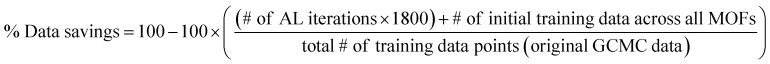


Based on the final GP models for all the MOFs, we achieved a data savings of 57.5% compared to the original MLP training data.

Fig. 2c illustrates the collective impact of AL on enhancing GP predictions across all 1800 MOFs, depicting the average *R*^2^ value (red line) at each iteration. In tandem with the improvement in *R*^2^, we present the corresponding percentage of data savings (blue line), as calculated by eqn (1). Notably, as AL iterations progress, we observe a consistent rise in the average *R*^2^ values, indicative of the AL criterion's efficacy. Around the 500th AL iteration and beyond, the average *R*^2^ across all GP models reaches 0.99, regardless of the GP mean uncertainty across MOFs. This trend underscores the potency of AL in optimizing dataset efficiency while upholding predictive accuracy, providing valuable insights for refining AL policies and strategies.

**Fig. 3:**
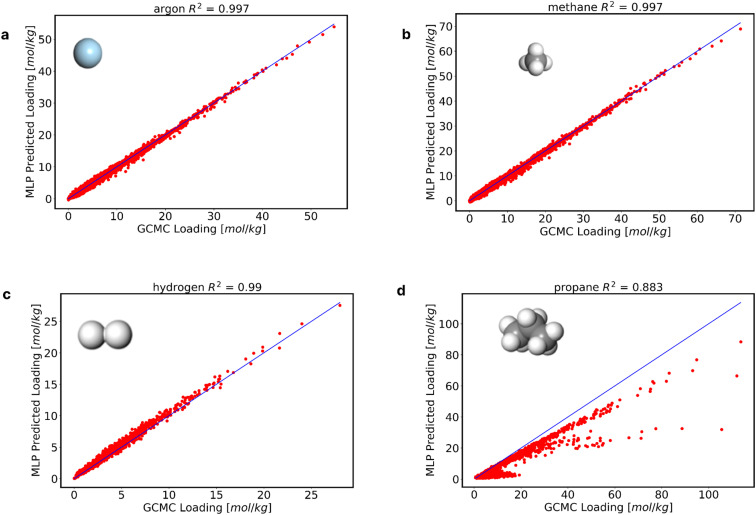
Perfomance of the newly developed MLP model. The new MLP model was evaluated in the prediction of adsorption of real molecules among the range of alchemical training adsorbates (a) argon, (b) methane, and extrapolated outside the range of alchemical training adsorbates (c) hydrogen, and (d) propane.

We developed 1800 GP models for MOFs using AL, necessitating a separate prediction case for each MOF when making predictions for other alchemical molecules. We leverage the datapoints used to train the final GP models at the 0.05 mol kg^−1^ uncertainty level for each of the 1800 MOFs to produce a new MLP model. All these datapoints were collected into one single training data featuring 2.1 million data points (57.5% data savings relative to the 5 million used to train the original MLP). Next, we utilized TensorFlow^43^ to train a new MLP model while optimizing its associated hyperparameters and a MAE loss function (details can be found in the ESI†), which was used to predict the adsorption of real molecules within different MOFs, as done previously by Gómez-Gualdrón and coworkers.^31^Fig. 3 shows a comparison between our GP-informed MLP and the GCMC simulations.

Notice that the predictions in Fig. 3 correspond to real molecules despite the training data corresponding to alchemical molecules. Note that molecules such as argon and methane (Fig. 3a and b, respectively) can be considered interpolations between alchemical molecules used in the training. The new MLP also retains the same limitations of the original MLP when predicting adsorption for real molecules that fall below and above the alchemical parameter “range” considered for training. While hydrogen predictions remain accurate (Fig. 3c), predictions for larger molecules like propane prove to be less successful (Fig. 3d). These results reemphasize the need to expand the training data to include, for instance, larger alchemical adsorbates, but we show that could be achieve efficiently using AL. Additional results and predictions can be found in the ESI.†

We further compared the predictions of the new MLP and the original MLP model. The results of these predictions of the adsorption of real molecules from both models are shown in Table 1. The new MLP model, trained on 57.5% less data than the original MLP model, exhibited a comparable performance to the original MLP model. A similar level of performance by both MLP models was maintained for molecules within and outside the alchemical range. These results show that AL is useful in scaling down on the training data required by MLP models. Specifically, for the same molecules in Fig. 3, we show the low-pressure region (0.02, 0.04, 0.06, and 0.08 bar) predictions in Fig. S7 of the ESI.† The model predicts the adsorption at the low-pressure regions fairly, with *R*^2^ values of 0.647, 0.777, 0.867 and 0.87 for argon, methane, ethane and xenon respectively.

**Table 1:** *R* ^2^ comparison between the original MLP model and new MLP model trained on the GP model final training data. The red items refer to molecules extrapolated outside the range of the alchemical training adsorbates

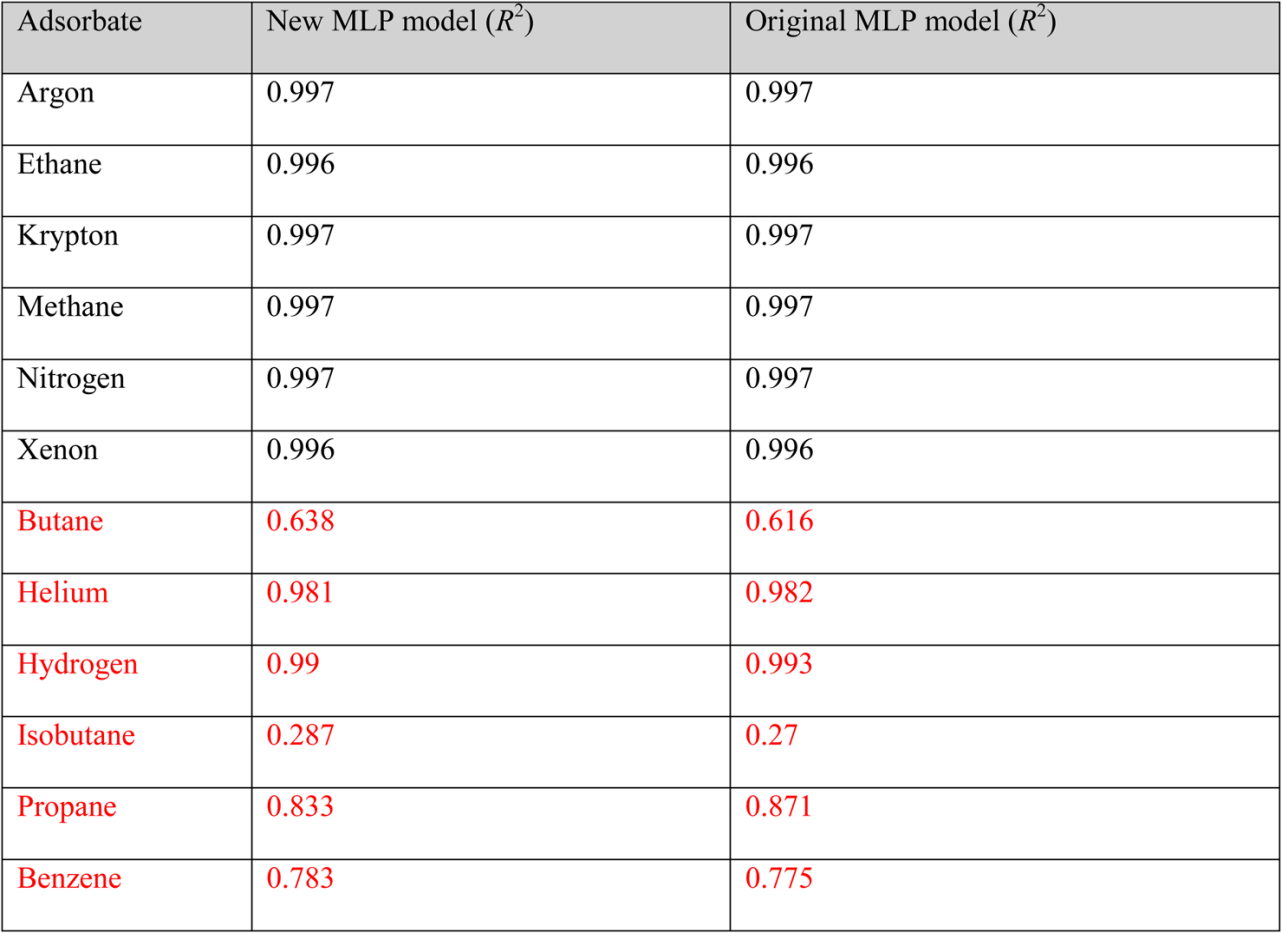

### Simultaneous AL on adsorbate, fugacity, and MOF space

The approximate halving of the training data by applying AL to the adsorbate space was encouraging, but arguably represents insufficient data savings for the increase in adsorbate space dimensionality that would occur if one expanded the types of adsorbates included in the training dataset to account for higher adsorbate complexity. Additionally, developing individual GP models for each MOF to generate the training data is notably demanding and tasking, as it requires performing AL on each MOF. To address this challenge, we decided to adopt a strategy where AL operates simultaneously on the adsorbate and MOF space. The underlying hypothesis was that what a model learns about adsorption in one MOF may be applicable to other similar MOFs, making exhaustive training data generation for all MOFs in a database unnecessary.

To achieve the above, we sought to incorporate the MOF features as additional input to a GP surrogate model within an AL framework that also selects the most “informative” MOFs to be used in training data generation. However, as the MOF space is inherently high-dimensional—where each MOF can be described as combination of chemical and textural characteristics such as node and linker types, void fraction, surface area, pore sizes, and so forth—it is imperative to reduce the dimensionality of the MOF representation to make AL exploration more efficient.

To this end, we resorted to principal component analysis^44–48^ (PCA), which is a widely employed technique to transform high-dimensional data into a lower-dimensional space, while retaining the essential patterns and structures inherent in the original data. In this study, PCA was applied to the textural properties of a larger number of MOFs (3445) than in our first approach. We used the following properties for PCA: largest pore diameter (LPD), diffusion-limiting pore diameter (DLPD), void fraction (VF), surface area (SA), standard deviation of the pore size distribution (PSSD), and the inverse framework density (IFD). These 3445 MOFs were the dataset MOFs from two previous studies.^31,42^ These MOF properties were chosen due to their deemed importance in predicting adsorption.^49–51^ The resulting principal components can be effectively interpreted as representative descriptors that capture the prevailing patterns and variabilities in the textural properties of the MOFs. Fig. S15† shows that the first two principal component directions (PC1 and PC2) account for 91% of the cumulative variance, suggesting they are sufficient for the AL to meaningfully navigate the MOF space. Formally, the inclusion of the MOF into the AL process makes it so that *N* ∼ *F*(*f*, *ε*, *σ*, *l*, *q*, PC1, PC2).

We selected four MOFs positioned at the boundaries of PC1 and PC2 to initiate the AL iterations. For each MOF, we provided adsorption data for a single alchemical adsorbate, chosen randomly, at a random fugacity. The initial training dataset comprises this data, while the remaining data from all the MOFs, fugacity, and adsorbates constitute the testing dataset in this context.

The bagging approach was applied to the full testing dataset. This approach involves the segmentation of the large dataset into bags of smaller datasets for predictions. The dataset, comprising 3445 MOFs and over 5 million simulation data points^31,42^ obtained through GCMC simulations, was systematically organized into 100 bags. Each bag was designed to contain diverse data samples, systematically categorized by fugacities and adsorbates. This categorization also ensured variability and comprehensive coverage within each set while incorporating all 3445 MOFs (represented by their PCs) in every bag. The purpose of the bagging process was simply to parallelize the testing of the model.

Upon segmentation, the initial GP model is evaluated on each bag, where the uncertainties in each data point are collected across all the bags. Upon compilation of uncertainties from the bags, the maximum GP uncertainty across all bags was estimated. At this point, the test array corresponding to adsorption at that specific point of maximum uncertainty was retrieved. Subsequently, this array, along with its corresponding GCMC adsorption data, was used to update the training dataset. This process was repeated 6000 times (see Fig. S17†).

**Fig. 4:**
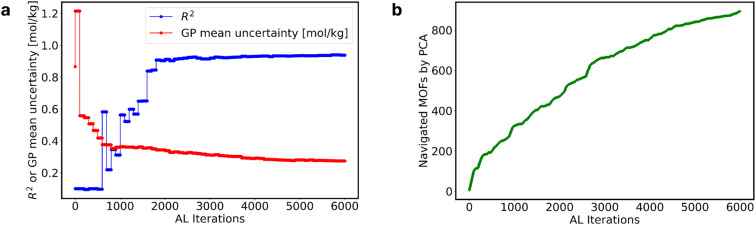
Analysis of the GP-PCA model. (a) Evolution of average *R*^2^, average GP MAE, and number of MOFs attained during 6000 iterations. (b) Number of MOFs navigated as a function of the number of iterations.

Upon 6000 iterations, the final training dataset selected by AL consists of 6004 data points. The evolution of the average *R*^2^ and GP mean uncertainty as a function of iteration is shown in Fig. 4a. Initially, the model had a GP mean uncertainty of 0.87 mol kg^−1^ and a low *R*^2^ of 0.1, which were substantially improved to a final average GP mean uncertainty of 0.27 mol kg^−1^ and an *R*^2^ of 0.94. Fig. 4b shows the number of MOFs used by the AL as the iterations increase, which by the 6000th iteration corresponds to 893 MOFs. Using just 0.11% of the data, encompassing only 26% of the MOFs in the database, we constructed a GP-PCA model with an *R*^2^ of 0.94. This kind of data savings are extremely encouraging for future exploration of datasets including a larger variety of adsorbate types.

We extended the AL iterations by an additional 1000 points, bringing the total to 7004 points. The final performance achieved an *R*^2^ of 0.941 and a mean absolute error (MAE) of 0.27 mol kg^−1^. This very minimal improvement in performance came at the cost of significantly increasing the amount of training data.

**Fig. 5:**
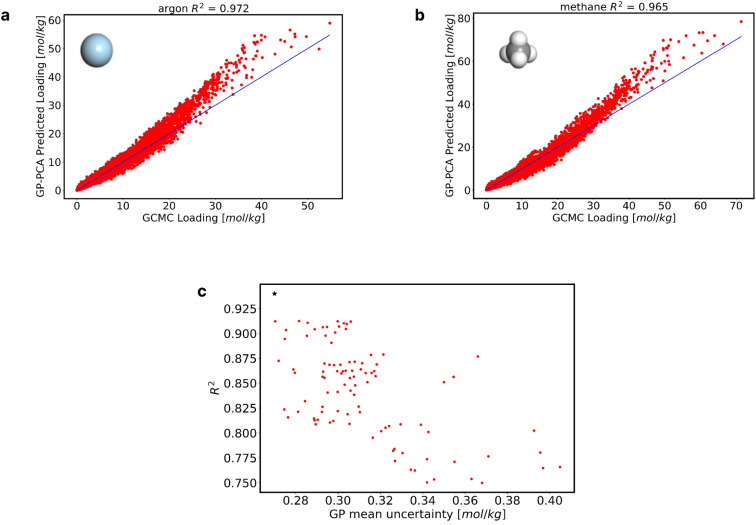
Performance of the GP-PCA model, and random sampled training data performance. (a) and (b) Model prediction of real molecules (argon, and methane). (c) *R*^2^(*s*) and GP MAE(*s*) across all randomly selected training data. Each point on the graph corresponds to a unique training dataset, with its associated *R*^2^ and GP MAE values.

Fig. 5a through 5b highlight predictions made for real molecules utilizing the newly developed GP model. Fig. 5a and b show adsorption predictions for argon and methane in the 3445 MOFs at a range of fugacities between 1 × 10^−2^ and 100 bar. Results for other real molecules are shown in Fig. S18.† With these results, it is possible to say that the model does well in predicting the adsorption of real molecules. In Fig. S19 of the ESI,† We also show the low-pressure adsorption (0.02, 0.04, 0.06, and 0.08 bar) performance of the GP-PCA model for the adsorbates in Fig. 5.

Notice that while we showed the possibility of training a suitable adsorption model using only 6004 datapoints, AL is directly responsible for that outcome. To illustrate this point, we fit GPs to 100 randomly selected training data sets, each with 6004 datapoints. Fig. 5c shows the significant variability in the average *R*^2^ and GP mean uncertainty for these 100 randomly selected training datasets. The lowest achieved *R*^2^ was 0.75, coupled with a GP mean uncertainty of 0.368 mol kg^−1^. Conversely, the highest *R*^2^ obtained was 0.91, accompanied by a GP mean uncertainty of 0.282 mol kg^−1^. On average, across all 100 randomly selected training datasets, the average *R*^2^ was 0.84, while the GP mean uncertainty averaged 0.312 mol kg^−1^, which are worse than the *R*^2^ of 0.94 and GP mean uncertainty of 0.27 mol kg^−1^ attained by the AL-selected dataset (star symbol in Fig. 5c).

Finally, we developed another MLP model trained using the 6004 data points from the GP-PCA model. Employing the same method and hyperparameters as previously applied, we fine-tuned the MLP model to ensure consistency and comparability with our earlier methodologies. Subsequently, we utilized this MLP model to predict the adsorption of real molecules across multiple MOFs. However, upon evaluation, our findings revealed a reduction in accuracy compared to the GP-PCA model. This divergence in predictive performance underscores the intricate challenges inherent in modeling gas adsorption phenomena within MOFs using traditional MLP approaches. For instance, employing the GP-PCA model to argon adsorption prediction yields an *R*^2^ value of 0.972, whereas the MLP model achieves an *R*^2^ value of 0.922. Other real molecules predictions can be found in the ESI (Fig. S23†).

### Feature navigation and evolution of the GP-PCA model

**Fig. 6:**
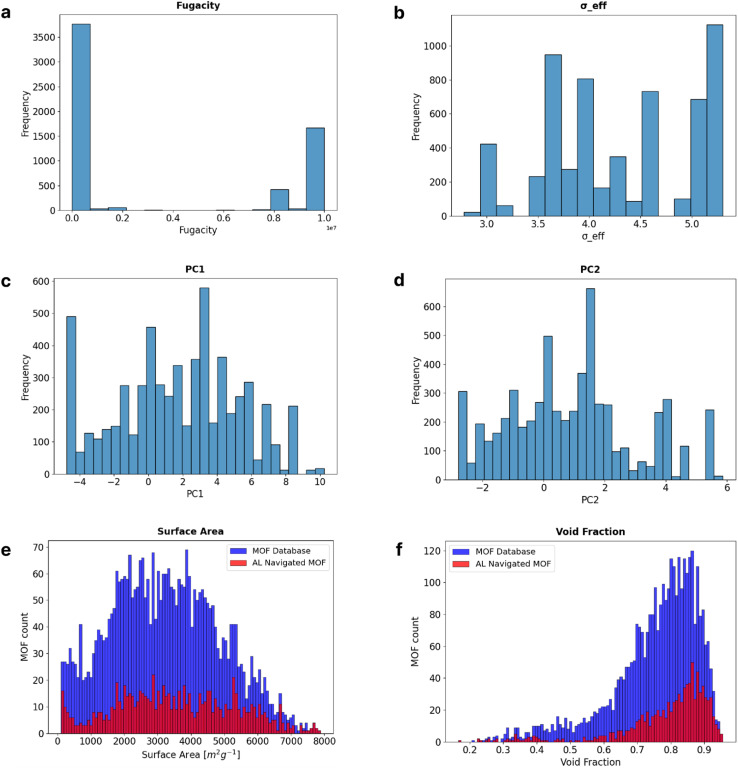
Training data sampled by the AL GP-PCA model. (a)–(d) AL selected training data regions for fugacity, effective sigma, PC1 and PC2, respectively. (e) and (f) AL sampled regions of surface area and void fraction as a comparison to the distribution of the available MOF dataset.

Looking to understand how AL made the selection of fugacity and adsorbate and MOF features to be included in the training dataset, Fig. 6a through 6d show the analysis of the some of the feature regions (fugacity, *σ*_eff_, PC1, and PC2). From the observations for fugacity, the model requires more training data at the boundaries of the feature. While it is not clear whether this is coincidental or not, it is worth noting that such fugacities tend to inform the model about adsorption at dilute (*i.e.*, Henry's region) and near-pore saturation conditions. Contrastingly, the model required more evenly distributed training data along the *σ*_eff_, PC1, and PC2 input features. From these results, we can see that AL intelligently selects what regions to sample and that it requires more diverse sampling for the adsorbates than any other input feature of the model. To get a more meaningful interpretation of the explored MOF space, we revert the PC1 and PC2 back to their textural properties, and in Fig. 6e and f, we show the regions navigated by the AL in terms of the surface area and void fraction. Remarkably, the observed distribution of textural properties in the 893 MOFs picked by AL mimics closely the distribution of these properties in the complete 3445-MOF set. This suggests that the AL picks a representative sample of MOFs for each combination textural property values.

## Summary and conclusions

AL has emerged as a critical methodology to optimize the data selection and acquisition for gas adsorption in MOFs and therefore to understand the most important features to predict their adsorption isotherms. Initially in this work, through a systematic application of AL to a dataset of 1800 MOFs, significant strides were made in reducing the dataset while continuously enhancing model performance. This iterative process was marked by a noticeable correlation between the reduction in GP mean uncertainty and the increment in *R*^2^ values through the iterative process, indicating a consistent improvement trend across multiple MOFs.

For the first approach with 1800 MOFs, we performed AL on each MOF by setting a convergence criterion of 0.05 mol kg^−1^ for the GP mean uncertainty, the resultant amalgamation of training data from diverse MOFs formed a comprehensive dataset encompassing approximately 2.1 million data points, which was used to train a new MLP model. This newly created MLP model showcased precision in predicting the adsorption behaviors of real molecules within the specified alchemical range. It was notable that the model's performance was comparable to the original one. This achievement highlights the efficiency of AL in selecting informative datasets while significantly saving computational resources.

To effectively extend our AL approach to new MOFs not included in the original dataset, it is crucial that these new materials share similar functional group densities and textural properties with the 1800 MOFs previously used in training the surrogate multilayer perceptron (MLP) model. This alignment is essential as it ensures the robustness of the AL process in selecting data points that effectively reduce model uncertainty, thereby preserving the accuracy and generalizability of the predictions. The initial dataset for these new MOFs must be carefully curated to encapsulate a comprehensive range of adsorption behaviors, enabling the AL algorithm to initiate an effective learning and optimization cycle. Thus, while our model demonstrates a significant capability in reducing data demands and efficiently predicting adsorption, its successful application to new MOFs hinges on the careful integration of these MOFs into the existing framework, ensuring that their properties do not deviate substantially from those within the original training set.

Furthermore, employing the same AL framework enables precise predictions of adsorption across different fugacities and adsorbates for these new, structurally and chemically similar MOFs, for which in this case the original MLP model can serve as the surrogate ground-truth model, ensuring accuracy and reliability in the prediction of adsorption properties.

While the MLP model demonstrates strong performance across various pressure ranges, the observed *R*^2^ values of 0.647, 0.777, 0.867, and 0.87 for argon, methane, ethane, and xenon at low pressures highlight inherent difficulties in accurately predicting adsorption at low loadings. These challenges suggest that the model's reduced performance in low-pressure regions may be due to the loss function (MAE) used, which might not adequately capture errors specific to these low-property areas.

To improve model accuracy in low-pressure regimes, future studies could explore alternative loss functions which may emphasize low-loading data regions, such as weighted mean squared error, mean absolute percentage loss and quantile loss.

As a second approach applied to 3445 MOFs, PCA was instrumental in identifying significant dimensions contributing to variance within MOF textural properties enhancing the MOF textural space exploration. Including PC1 and PC2 in the AL process contributed to robust model training and enhanced predictive accuracy. Furthermore, an in-depth analysis of textural properties within the navigated MOF subset shows the preservation of the overall distribution of textural properties compared with the available data. This diverse and extensive coverage across MOF textural properties is observed in the comparative histograms. This fact is proof of the navigation process's effectiveness in encapsulating and representing essential material characteristics within the navigated subset.

In our second approach, the successful application of this approach to new MOFs presents a challenge, primarily in ensuring that the PCA accurately represents these additional MOFs. It is essential that the textural properties of new MOFs do not deviate significantly from those encapsulated by the original PCA model used in training. If new MOFs introduce substantially different features, the PCA model might require recalibration or expansion to include these new dimensions. Such adjustments will ensure that the model maintains its reliability and continues to offer precise adsorption predictions as it is extended to include a wider variety of MOF structures.

To enhance the applicability of our GP-PCA model and address MOFs that fall outside the current scope of this study (MOFs that have their textural properties outside the range of MOFs in this study), the dataset can be expanded to include a broader range of MOFs with diverse textural properties. By incorporating these MOFs, we can improve the model's robustness and predictive accuracy.

The scope of this work is primarily constrained by the limited range of considered adsorbates, delineated by the parameters of the alchemical model. However, this constraint mirrors that of previous models. Specifically, the study focuses on predicting adsorption isotherms for small, nearly spherical, nonpolar, monoatomic, and diatomic adsorbates across various fugacities, at 298 K, consistent with the conditions of GCMC simulations. Despite these limitations, the integration of AL in data generation represents a significant advancement toward establishing a more comprehensive and universally applicable adsorption model for gases within MOFs. This approach signifies progress in refining predictive models, particularly in terms of reducing data requirements. Importantly, it sets a clear path for expanding the model's versatility by incorporating new data, thereby enhancing its applicability across a broader range of scenarios.

In summary, these cumulative findings highlight the efficacy of AL in navigating complex MOF and adsorbate spaces, accurately predicting adsorption, and enriching our understanding of the phenomena, specifically in MOFs. This evidence solidifies AL as a valuable and necessary methodology in material science research, offering an effective way to overcome data-scarcity while paving the way for future advancements in this domain.

In future studies, we hope to use AL to navigate even more complex scenarios like alchemical mixture adsorptions. In a previous study, Mukherjee and coworkers showed that they can use AL to make predictions of three different sets of binary gases varying pressure, composition, and temperature simultaneously. For low pressure regions, they also showed that the Ideal Adsorbed Solution Theory (IAST) works well in predicting mixture adsorption from pure component adsorptions.^30^ Also, Gómez-Gualdrón and coworkers showed that that IAST can predict binary adsorption for mixtures at multiple compositions and pressures.^42^ With these tools, we are confident that we would be able to navigate the complex alchemical mixture space.

Also, the AL methodology demonstrated in this paper with MOFs holds immense potential for broader applications to other porous materials. Due to their vast structural and chemical diversity, MOFs encompass a wide array of pore types and chemical compositions that are representative of most adsorbents. This diversity positions MOFs as an ideal model system, suggesting that the AL approach can be effectively extended to optimize and predict adsorption properties across a wide spectrum of porous materials.

Finally, it is worth mentioning that in this study, the alchemical concept is applied solely to the adsorbates and not the MOFs. However, previous research by Fanourgakis and coworkers^52^ has demonstrated one of the potential ways of alchemical modifications in MOFs by artificially adjusting the interaction parameters of framework components, such as linkers and nodes, to enhance their adsorption properties. Fanourgakis and coworkers^52^ introduced these MOFs by modifying the sizes of framework atoms and increasing the interaction strength between the framework and guest molecules (Lennard-Jones parameters). This approach highlights the broader utility of alchemical modifications in enriching the dataset for AL driven models and enhancing their predictive power.

In this work, we have focused exclusively on alchemical adsorbates to streamline the model development and validation process, ensuring a clear and controlled exploration of the adsorbate space. In future studies, we intend to extend our approach to include both alchemical adsorbates and MOFs, thereby leveraging the full potential of alchemical modifications to further refine and expand the capabilities of ML models in predicting adsorption.

## Code availability

The AL algorithms, along with examples of the GP model and the newly developed MLP model tailored for fugacity and adsorbate scenarios, are accessible on GitHub. Additionally, both the GP-PCA and MLP models, designed to encompass fugacity, adsorbate, and adsorbent space, are available on GitHub at https://github.com/theOsaroJ/Active-Learning-of-alchemical-adsorption-simulations-towards-a-universal-adsorption-model.

## Author contributions

The authors confirm contributions to the paper as follows: study conception and design: Y. J. C., D. A. G-G., E. O., F. F. R.; methodology: Y. J. C., D. A. G-G., E. O.; code development and execution: E. O., G. M. C.; simulations: F. F. R., E. O.; writing – initial draft: E. O.; writing – review & editing: Y. J. C., D. A. G-G., E. O., F. F. R.; supervision & funding: Y. J. C., D. A. G-G.

## Conflicts of interest

There are no conflicts to declare.

## Supplementary Material

SC-015-D4SC02156H-s001

## Data Availability

The data used in this study was obtained from two previous studies.^31,42^ However, the data was reorganized to be used for this research study. The reorganized data can be found on the GitHub page *via*: https://github.com/theOsaroJ/Active-Learning-of-alchemical-adsorption-simulations-towards-a-universal-adsorption-model.
