# Supplementary material for: Active learning of alchemical adsorption simulations; towards a universal adsorption model
Source: Chem Sci. 2024 Oct 8;15(42):17671–84. doi: 10.1039/d4sc02156h (PMC11459438; doi:10.1039/d4sc02156h)
Supplement: SC-015-D4SC02156H-s001 [file SC-015-D4SC02156H-s001.pdf]

Supplementary Information (SI) for Chemical Science.
This journal is © The Royal Society of Chemistry 2024

SUPPORTING INFORMATION

Active learning of alchemical adsorption simulations; towards a universal adsorption model.

Etinosa Osaro[a], Fernando Fajardo-Rojas[b], Gregory M. Cooper[a], Diego Gómez-Gualdrón[b], and Yamil J. Colón*[a].

a. Department of Chemical and Biomolecular Engineering, University of Notre Dame, IN 46556, USA
b. Department of Chemical and Biological Engineering, Colorado School of Mines, 1500 Illinois St, Golden, CO 80401, USA

*Corresponding author: Yamil J. Colón (ycolon@nd.edu)

Table of Contents

The Alchemical Features 3

Test data range for fugacity and adsorbate space 3

Active Learning policy choice evaluation 5

Effects of MOFs textural properties on AL iterations on the fugacity and adsorbates space. 6

Hyperparameter Tuning of the new MLP model. 9

New MLP adsorption prediction of real molecules 10

Effects of textural properties and fugacity on the new MLP model predictions. 12

Principal Component Analysis of MOFs 20

Active Learning Procedure for simultaneous navigation of fugacity, alchemical, and MOF space. 21

Feature Navigation by GP-PCA model 24

MLP model developed from GP-PCA training data. 26

Glossary of Machine Learning Jargon. 28

The Alchemical Features

i. The effective ε (eps_eff) parameter characterizes the depth of the potential energy well, indicating the strength of attraction between the adsorbate atoms.

ii. The effective σ (sig_eff or _eff) parameter defines the distance at which the potential energy between the adsorbate atoms is zero, representing the size of the adsorbate molecule.

iii. Bond Length (l) which refers to the length of bonds within the adsorbate molecule.

iv. Charge (q) which represents the net electrical charge carried by the adsorbate molecule.

Test data range for fugacity and adsorbate space

The ranges of the test data used in this study are shown in table S1 below. Various combinations of these parameters were generated, and the full dataset can be found in the project GitHub repository (https://github.com/theOsaroJ/Active-Learning-of-alchemical-adsorption-simulations-towards-a-universal-adsorption-model) under the fugacity and adsorbate space folder.

Table S1. Parameters for the test alchemical adsorbates; all possible combinations were considered.

Fugacity	eps_eff	sig_eff	chg	bond_length
1000	30	3	0	0
3350	55	3.6	0.3	1.2
5750	65	3.75	0.5	1.5
8250	95	3.9	0.9	1.6
15000	105	4.1		
33500	125	4.3		
57500	155	4.45		
82500	195	4.6		
150000	205	5.1		
575000	250	5.25		
1500000		5.3		
8250000				
10000000				

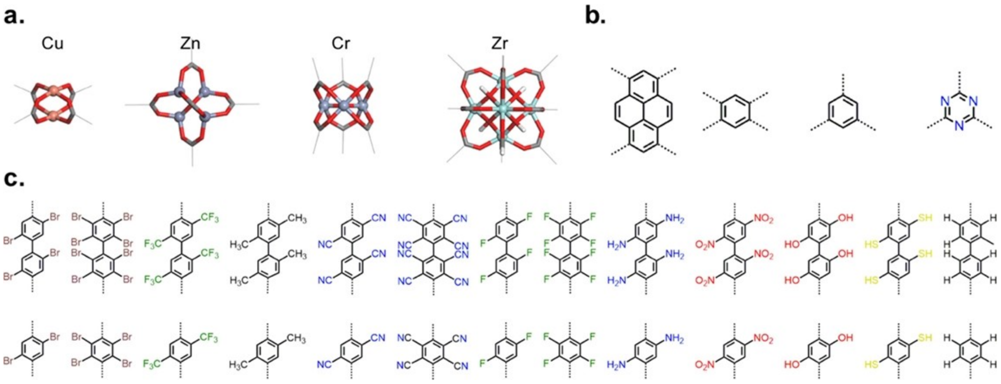

Figure S1. **Components Utilized in Constructing the MOF Database:** This figure illustrates the building blocks employed in assembling the MOF database. The dashed lines represent points of connection to the remainder of the framework. It includes: (a) inorganic nodes featuring metal-containing elements such as Cu (4-connected), Zn (6-connected), Cr (6-connected), and Zr (8- and 12-connected) oxoclusters; (b) organic nodes which are the central segments of multitopic organic linkers; and (c) connecting components which consist of the extensions of multitopic organic linkers or the main structure of ditopic linkers. Figure is adapted with permission from Ref. 31 in manuscript, Copyright 2020, American Chemical Society.

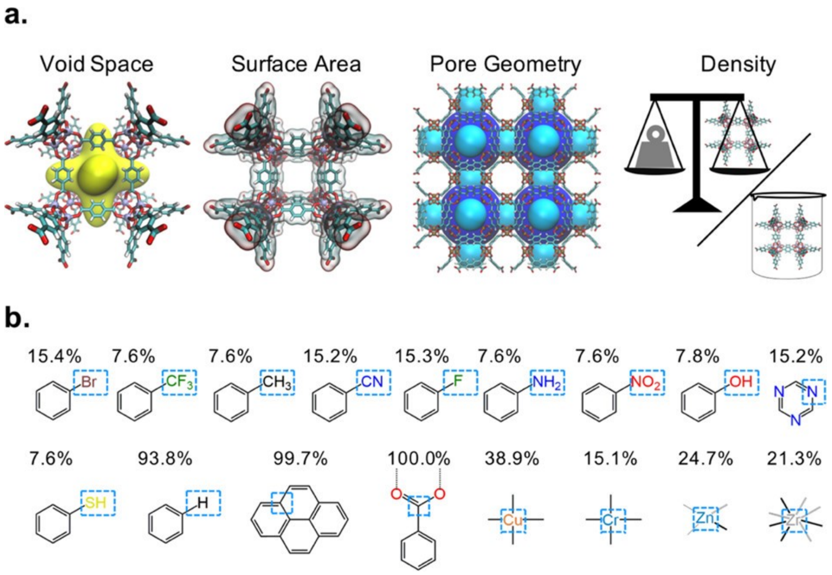

Figure S2. Descriptors Used to Create the MOF Fingerprint: This figure details the specific descriptors that make up the MOF fingerprint. (a) Six textural properties are included: void fraction, gravimetric surface area, largest pore diameter (LPD, represented by dark blue spheres), pore limiting diameter (PLD, represented by light blue spheres), pore size standard deviation (PSSD), and density. (b) Seventeen chemical motifs are outlined (boxed), with calculations made for the number density of each motif within each MOF. Additionally, the percentage of MOFs within the database that contain each motif is displayed at the top. Figure is adapted with permission from Ref. 31 in manuscript, Copyright 2020, American Chemical Society.

Active Learning policy choice evaluation

To evaluate the AL policy, which underpins this entire study, we conducted two experiments: (a) using a GP mean uncertainty threshold of 0.05 mol/kg, and (b) employing a GP maximum relative error threshold of 2%. These experiments were carried out on 10 MOFs, including 5 with the lowest surface areas and 5 with the highest surface areas. The results indicated that the GP mean uncertainty threshold of 0.05 mol/kg provided greater accuracy on average and required fewer sampled points compared to the GP maximum relative error policy. These results are shown in table S2.

Table S2: Performance of 10 distinct MOFs model based on two AL policies.

MOF Name	Surface Area [m2/g]	Model R^2 (chosen policy)	Final Training Data (chosen policy)	R^2 (relative error policy)	Final Training Data (relative error policy)
OPT_mrc_sym_6_mc_3_1AN_C_1B_4CN_ntn_edge	105.716807	0.99	1558	0.97	1514
OPT_sqca_sym_8_mc_9_sym_3_on_2_ntn_edge_ntn_edge	107.565698	0.99	1564	0.99	1587
OPT_mrc_sym_6_mc_3_1AN_C_1B_4Br_ntn_edge	116.574471	0.99	1567	0.99	1684
OPT_lim_sym_5_mc_2_sym_3_on_2_1B_2OH_ntn_edge	117.752316	0.99	1445	0.98	1571
OPT_fcu_sym_12_mc_11_1B_2NO2	122.458283	0.99	1541	0.99	1562
OPT_ucp_sym_5_mc_2_sym_3_on_2_2B_4H_2B_4H	7629.816166	0.99	652	0.99	983
OPT_stu_sym_5_mc_2_sym_3_on_2_2B_4H_2B_4H	7656.57517	0.99	1196	0.99	1272
OPT_stx_sym_5_mc_2_sym_3_on_2_2B_4H_2B_4H	7677.384874	0.99	1071	0.99	1069
OPT_lnj_sym_3_on_2_sym_7_mc_4_2B_4H_2B_4H	7756.190498	0.98	740	0.99	1006
OPT_fog_sym_5_mc_2_sym_3_on_2_2B_4H_2B_4H	7763.379427	0.99	507	0.99	981

Effects of MOFs textural properties on AL iterations on the fugacity and adsorbates space.

As seen in **Figure S3a and S3b**, we analyze the relationship between the number of AL iterations and the structural features. In **Figure S3a**, we show the number of final AL iterations for all MOFs in terms of their textural features which includes Largest Pore Diameter (LPD), Pore Limiting Diameter (PLD), Surface Area (SA), Void Fraction (VF), Pore Size Standard Deviation (PSSD) and Inverse Framework Density (IFD). In **Figure S3b,** the Principal Components (PC) plot visualizes the first two principal components, capturing the most significant variance in the data. The Red points represent structures with iterations > 1000, while blue points represent structures with iterations ≤ 1000. The Red points are more densely clustered within specific regions of the PCA plot, indicating a certain homogeneity in their structural features.

To further investigate the features in terms of the PCs, we computed the PC factor loading which represents the coefficients or weights of the original variables (features) in the principal components. They indicate how much each original variable contributes to each principal component. From the second panel in figure S3b, we observe that PC1 is strongly influenced by LPD, PLD, IFD, and VF, as indicated by their high factor loadings; and PC2 is influenced significantly by PSSD (positively) and SA (negatively). Structures with high iterations (> 1000) tend to have lower values for SA, IFD, and VF, which are key features contributing to PC1 and PC2.

In summary, MOFs requiring more than 1000 iterations tend to have lower values for SA, IFD and VF. They also cluster within specific regions in the PCA plot, indicating homogeneity in these structural features.

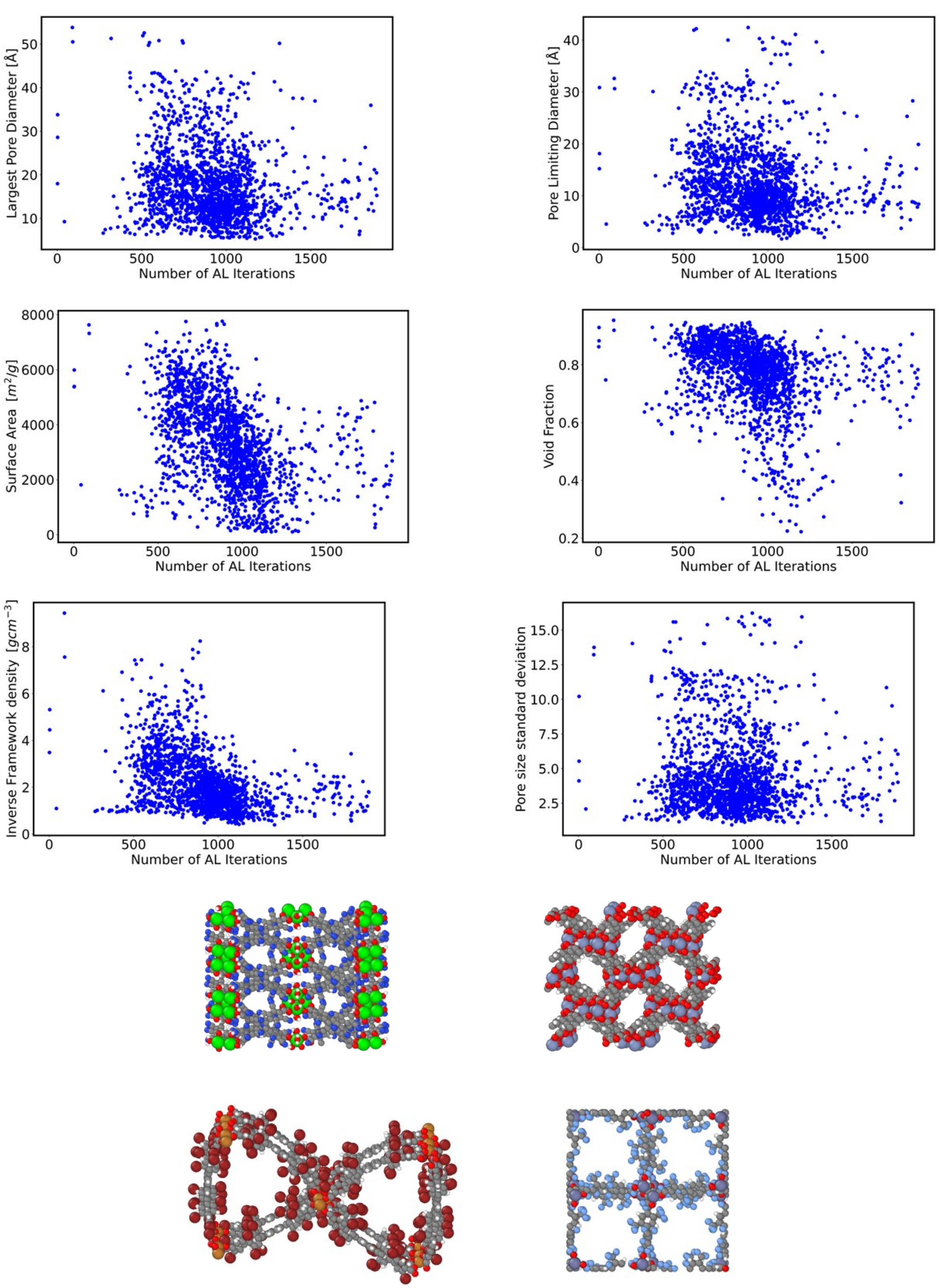

Figure S3a. First Six Images: Influence of MOF textural properties on the AL iterations required to achieve 0.05mol/kg GP mean uncertainty. First MOF (OPT_csq_sym_5_on_9_sym_8_mc_9_2B_4Br) with high AL iterations. Second MOF (OPT_ucp_sym_5_mc_2_sym_3_on_2_2B_2CH3_2B_2CH3) with low AL iterations. Third MOF (OPT_csq_sym_5_on_9_sym_8_mc_9_1B_4CN) with highest R^2. Last MOF (OPT_dag_sym_3_on_2_sym_7_mc_4_ntn_edge) with the lowest R^2. MOFs are from left to right.

To further analyze the effects of textural properties on the number of AL iterations, we transformed all the structural features to Principal Components and their space as seen in **Figure S3b** below:

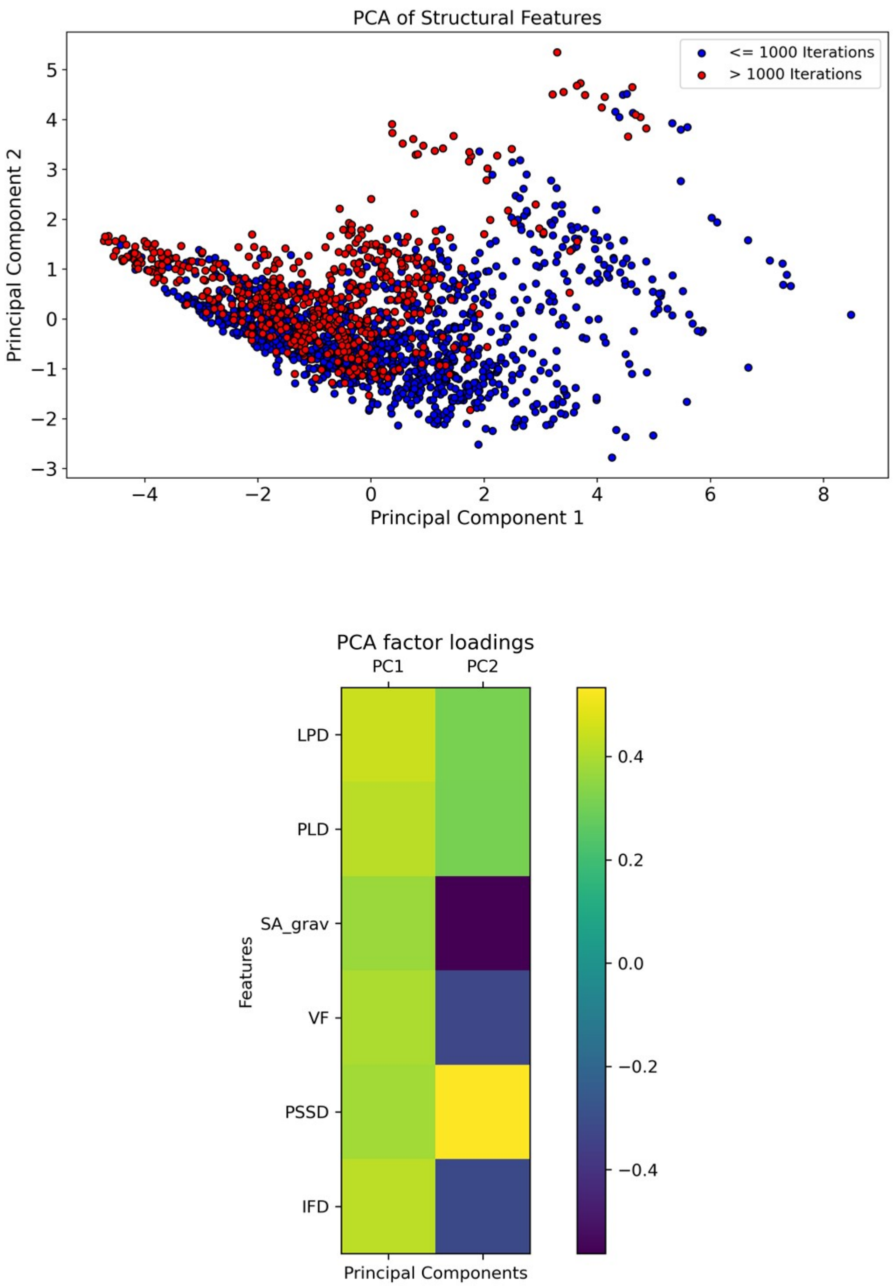

Figure S3b. First Panel: PCA visualization of MOFs in terms of high and low AL iterations. Each point represents the PC equivalent of all textural properties of a single MOF. Second Panel: The PCA factor loadings of all textual properties in the PC space.

Hyperparameter Tuning of the new MLP model.

The hyperparameters tuned for the development of the new MLP model based on the final cumulative AL training data across all MOFs are shown in the table below.

Table S3: Hyperparameters list to be tuned.

Epochs	Batch Size	Learning Rate
100	32	0.001
200	64	0.0001
500	128	0.00001

Based on the loss (MAE), the best model was developed with 500 epochs, a batch size of 128 and a learning rate of 0.00001. The resulting loss plot (learning curve) is shown below.

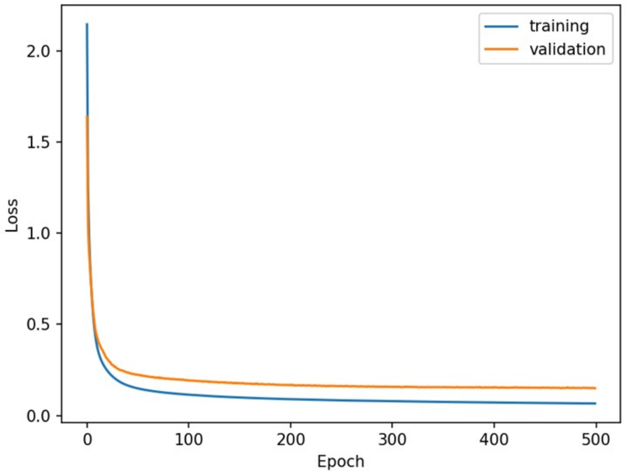

Figure S4: Learning curve (MAE) for best model from the hyperparameter tuning list.

New MLP adsorption prediction of real molecules

The new MLP model was used to make predictions of real molecules within and outside (extrapolation) the alchemical feature range. The plots are shown below.

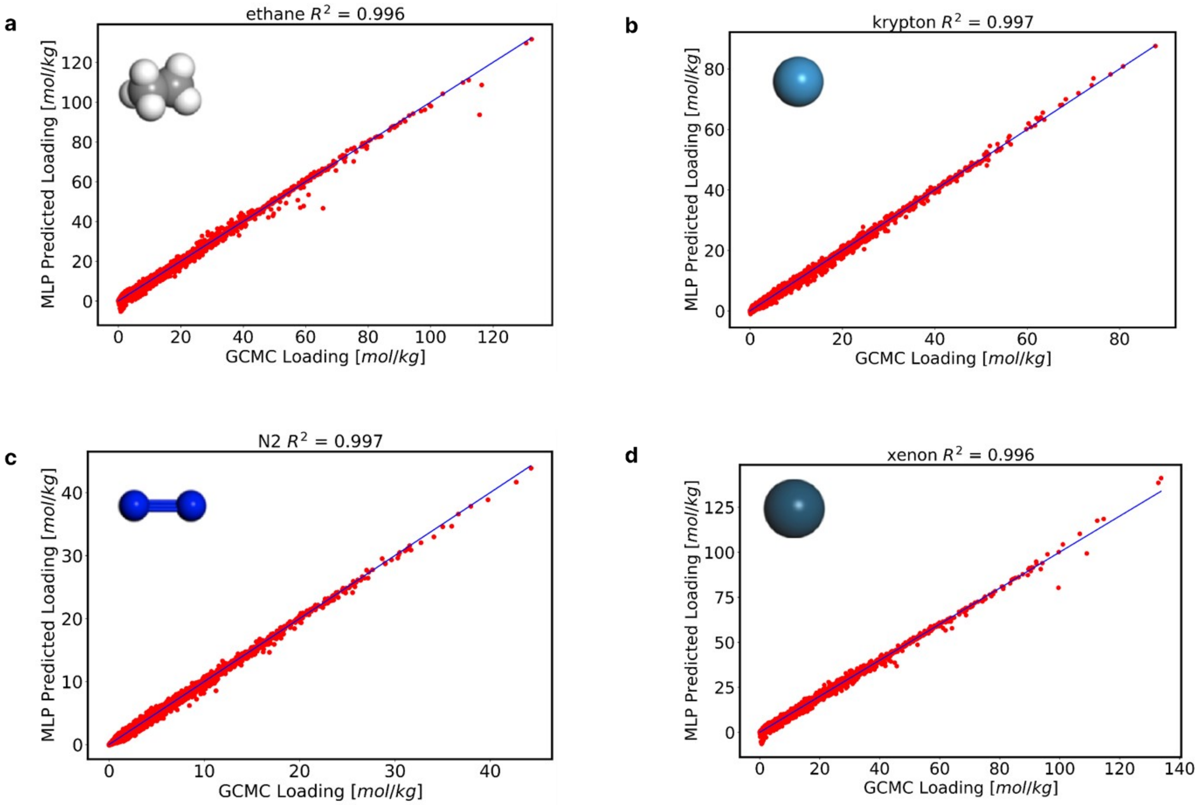

Figure S5: Comparison of MLP model generated from the GP model priors versus GCMC loading across MOFs. These molecules have their adsorbate features within the alchemical ranges used during the model training.

The figures below show the predictions of adsorption of molecules outside the alchemical training data range.

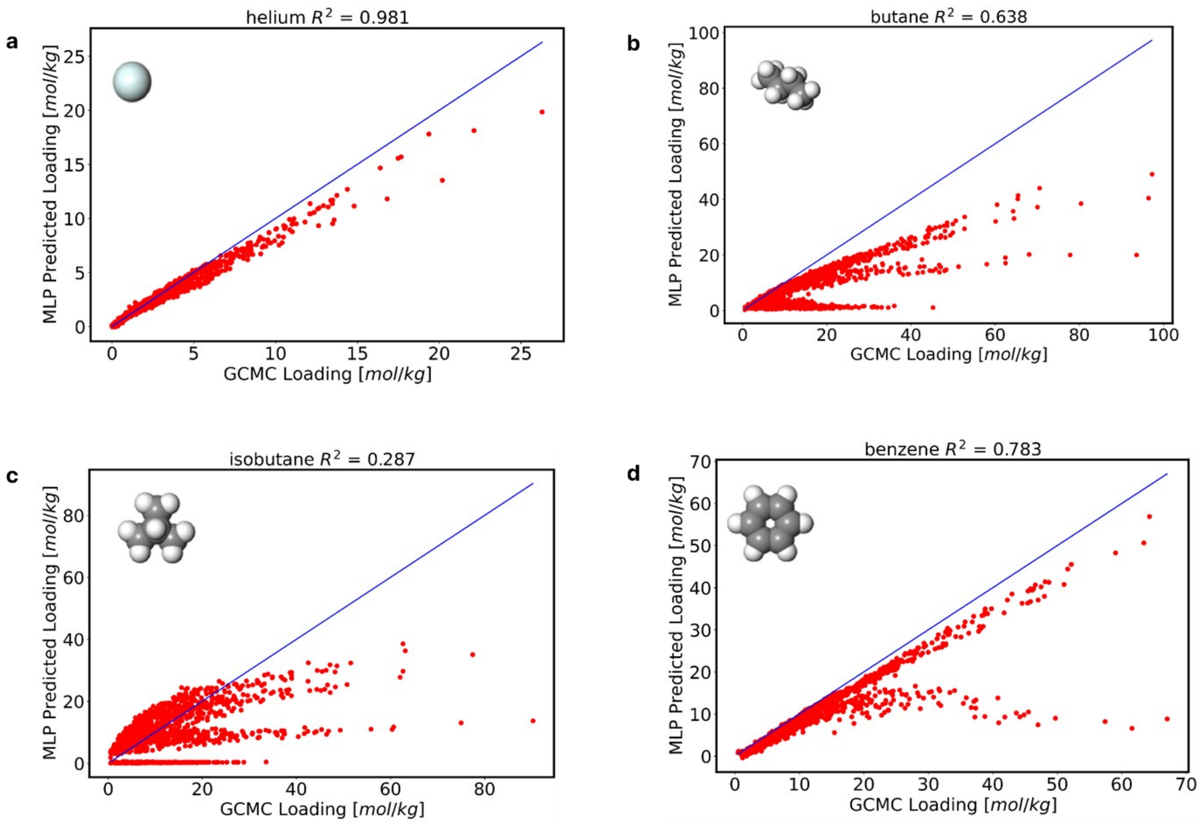

Figure S6: Comparison of MLP model generated from the GP model priors versus GCMC loading across MOFs. These molecules do not have their adsorbate features within the alchemical ranges used during the model training.

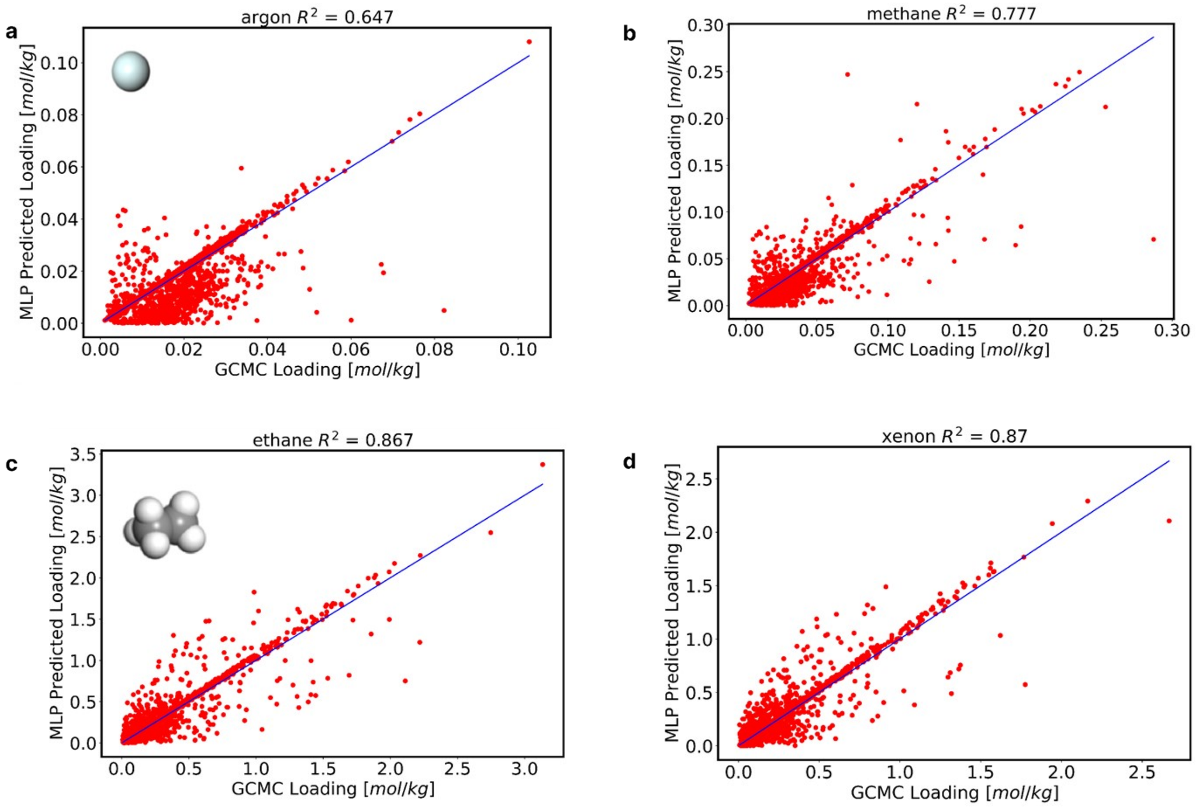

Figure S7: Comparison of MLP model generated from the GP model priors versus GCMC loading across MOFs, **at low pressures(≤ 0.08bar)**. These molecules have their adsorbate features within the alchemical ranges used during the model training.

Effects of textural properties and fugacity on the new MLP model predictions.

When extending predictions beyond the alchemical range, we observed lower R^2 values, highlighting the challenge of extrapolation due to inherent uncertainty outside the known data domain. The material's textural properties significantly influence the accuracy of the MLP model, especially in extrapolating adsorption behavior for molecules like propane, butane, isobutane, and benzene. These molecules, falling beyond the trained alchemical range, serve as valuable examples to understand how material properties impact the model's predictive capacity during extrapolation.

From the inspections in the figures below, at higher void fractions around the 0.9 range (see figure S14) within these MOFs, the predictive accuracy of the model regarding adsorption behavior may decrease due to several underlying reasons. Elevated void fractions typically imply larger empty spaces within the MOF structure, leading to fewer available interaction sites for adsorbate molecules. Consequently, molecules outside the trained alchemical range may exhibit less accurate predictions due to limited interaction possibilities. Moreover, the abundance of voids might reduce available adsorption sites within the MOF, particularly affecting the adsorption capacities of larger molecules like propane, butane, isobutane, or benzene. This limitation can result in inaccuracies when predicting their adsorption behavior. Additionally,

while voids contribute to overall porosity, they might not offer significant surface areas for effective MOF-adsorbate interaction.

We also observed that the MLP model used on the extrapolated molecules is not able to accurately predict the adsorption at low fugacities as clearly shown in figure S17.

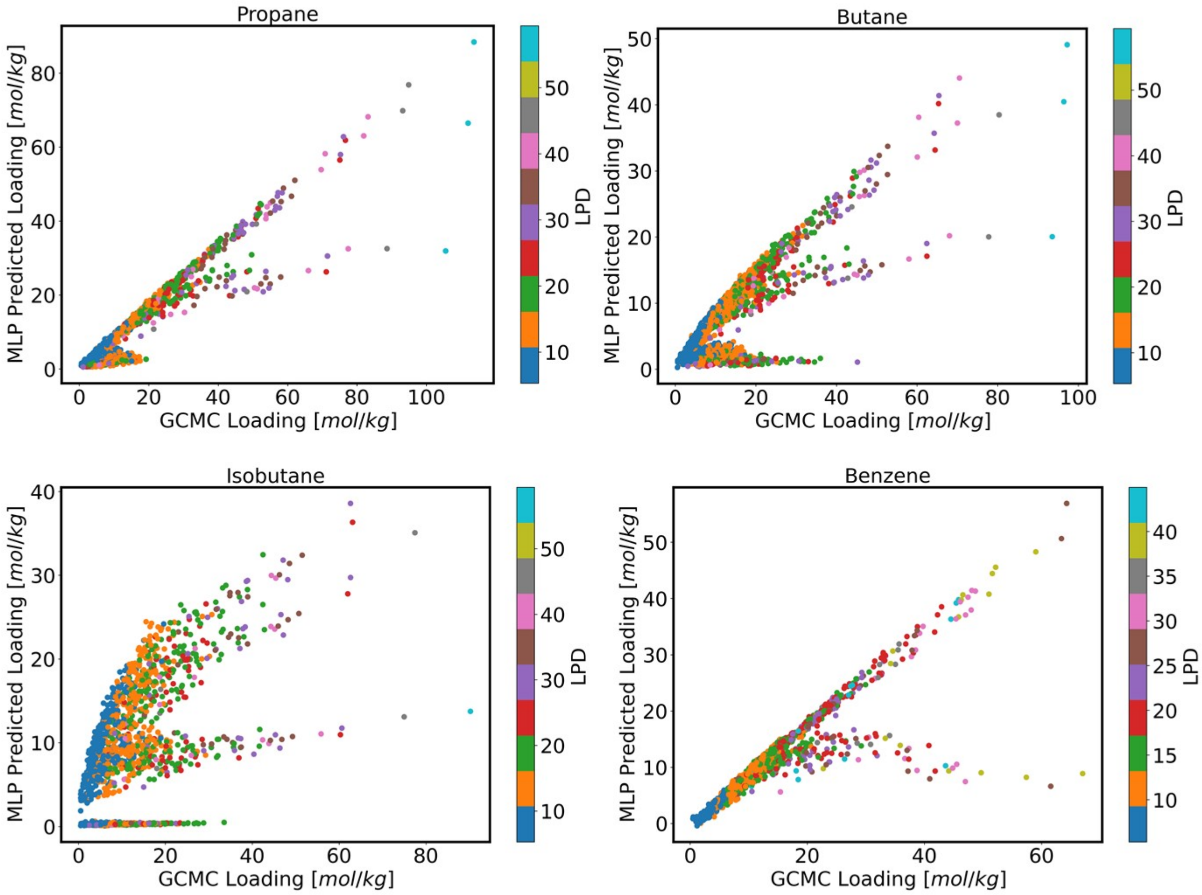

Figure S8. Illustration showcasing the influence of largest pore diameter [Å] on the predictive accuracy of adsorption behavior in MOFs.

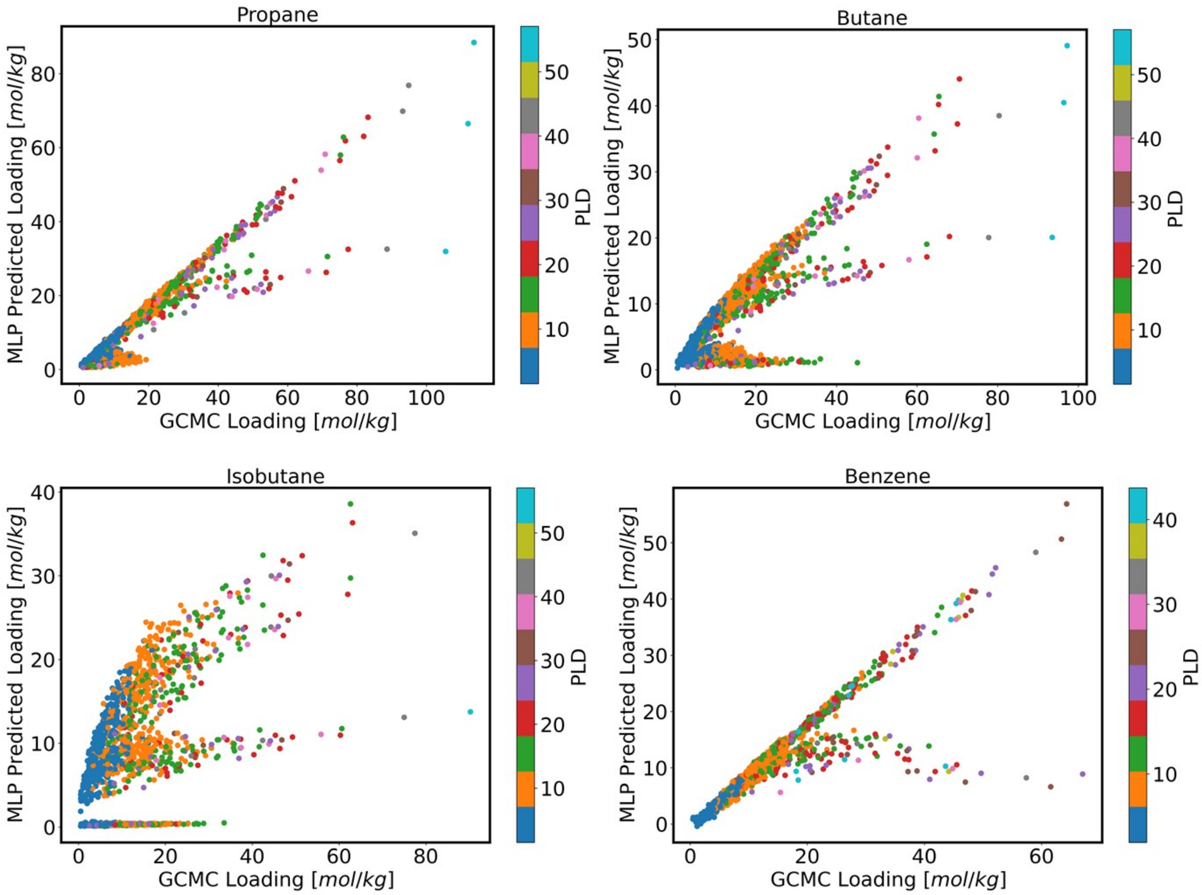

Figure S9. Illustration showcasing the influence of Pore limiting diameter [Å] on the predictive accuracy of adsorption behavior in MOFs.

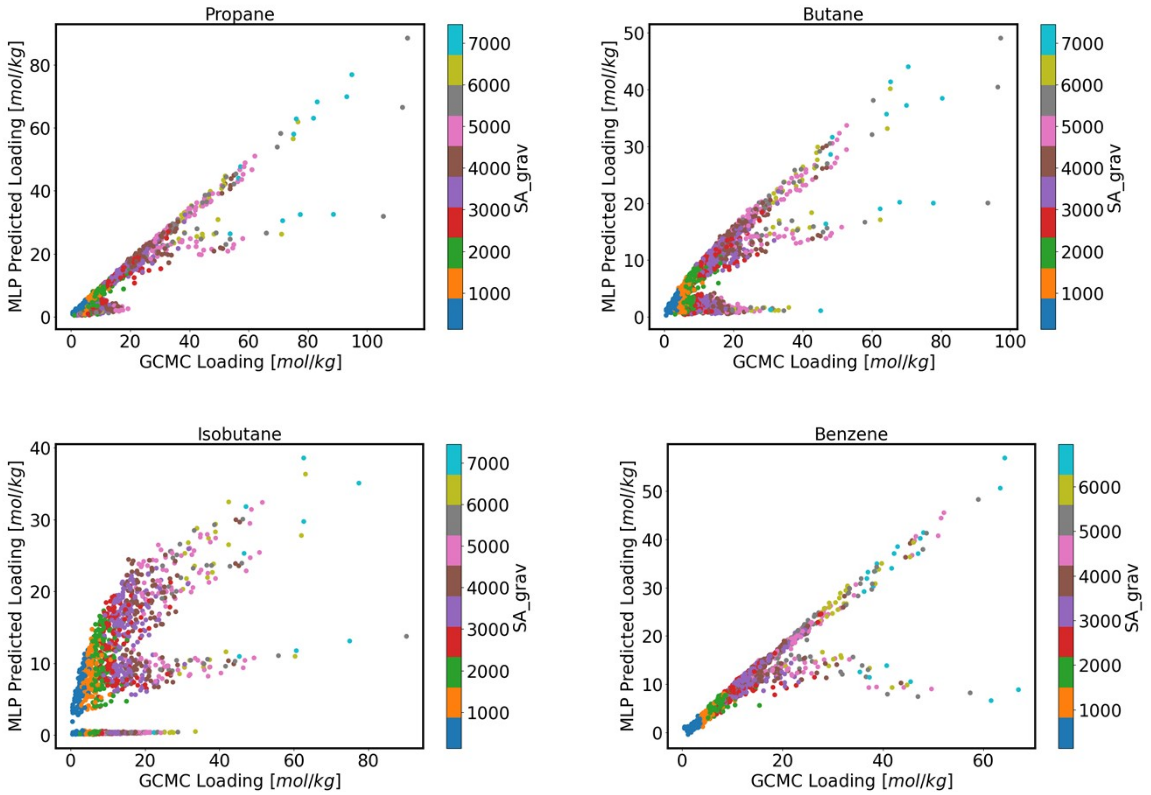

Figure S10. Illustration showcasing the influence of surface area [m^2g^{-1}] on the predictive accuracy of adsorption behavior in MOFs.

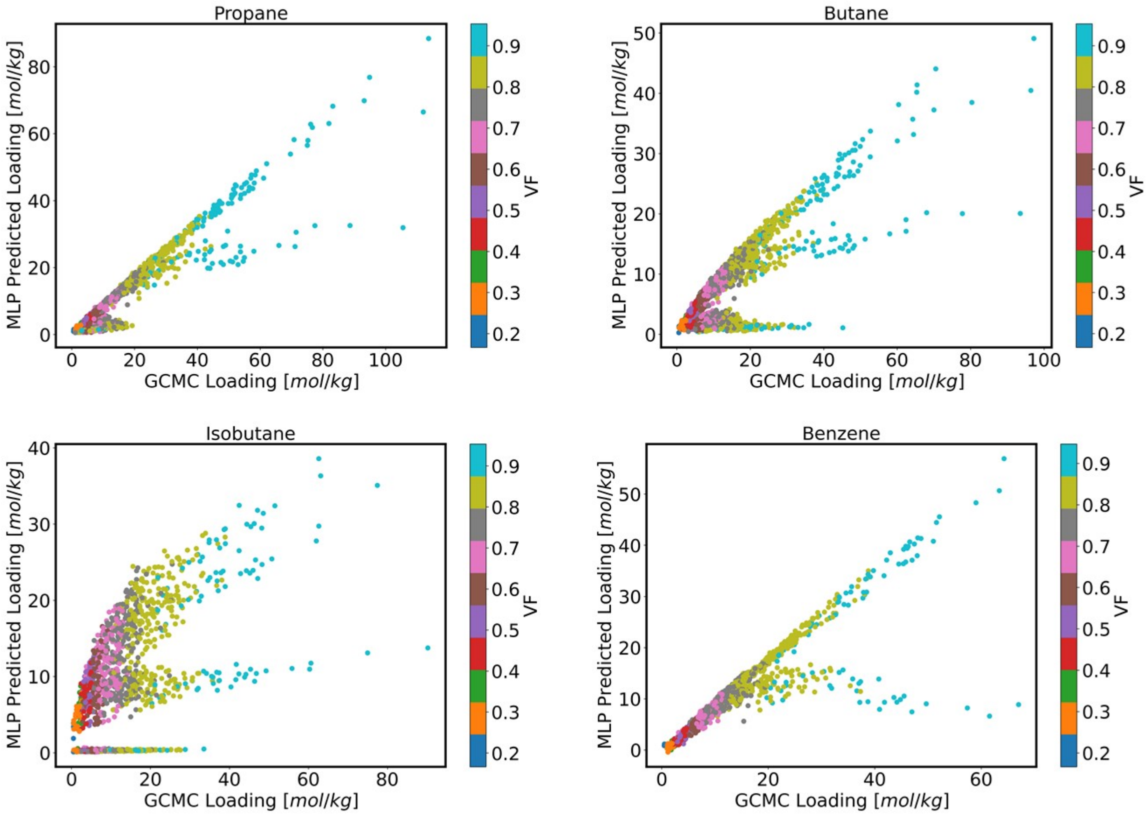

Figure S11. Illustration showcasing the influence of void fraction on the predictive accuracy of adsorption behavior in MOFs.

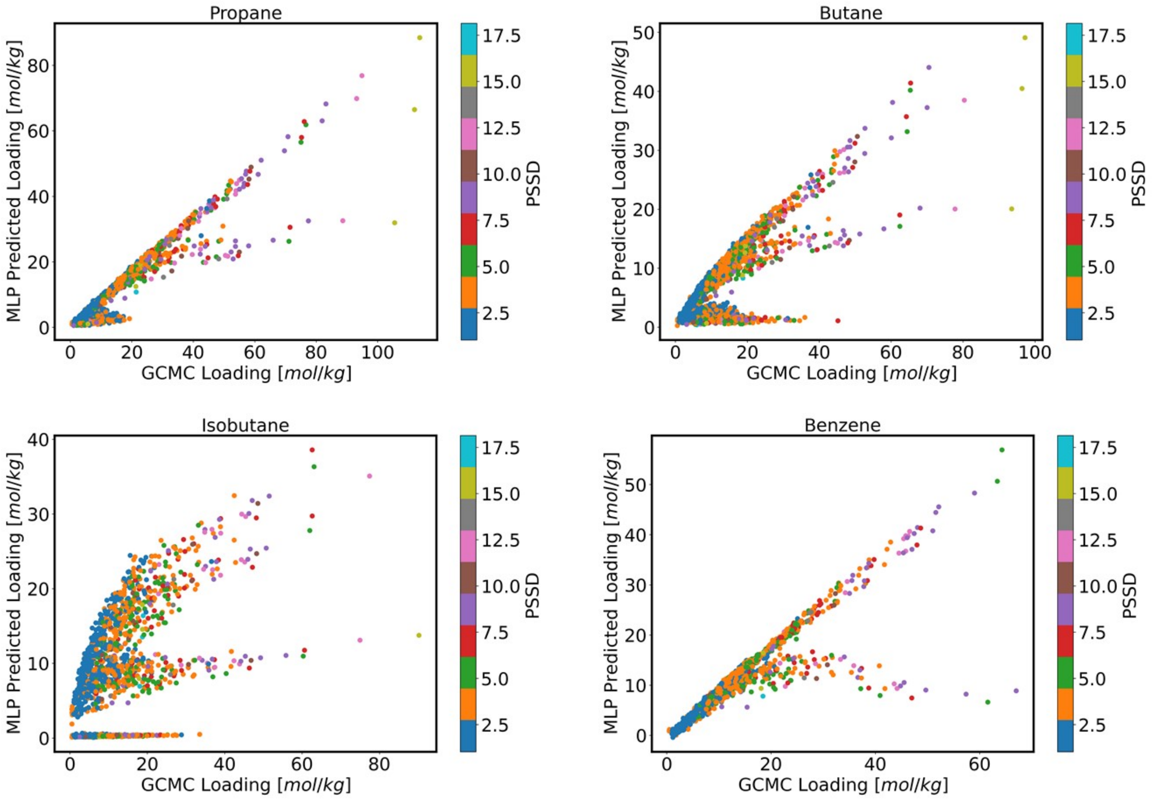

Figure S12. Illustration showcasing the influence of pore size standard deviation on the predictive accuracy of adsorption behavior in MOFs.

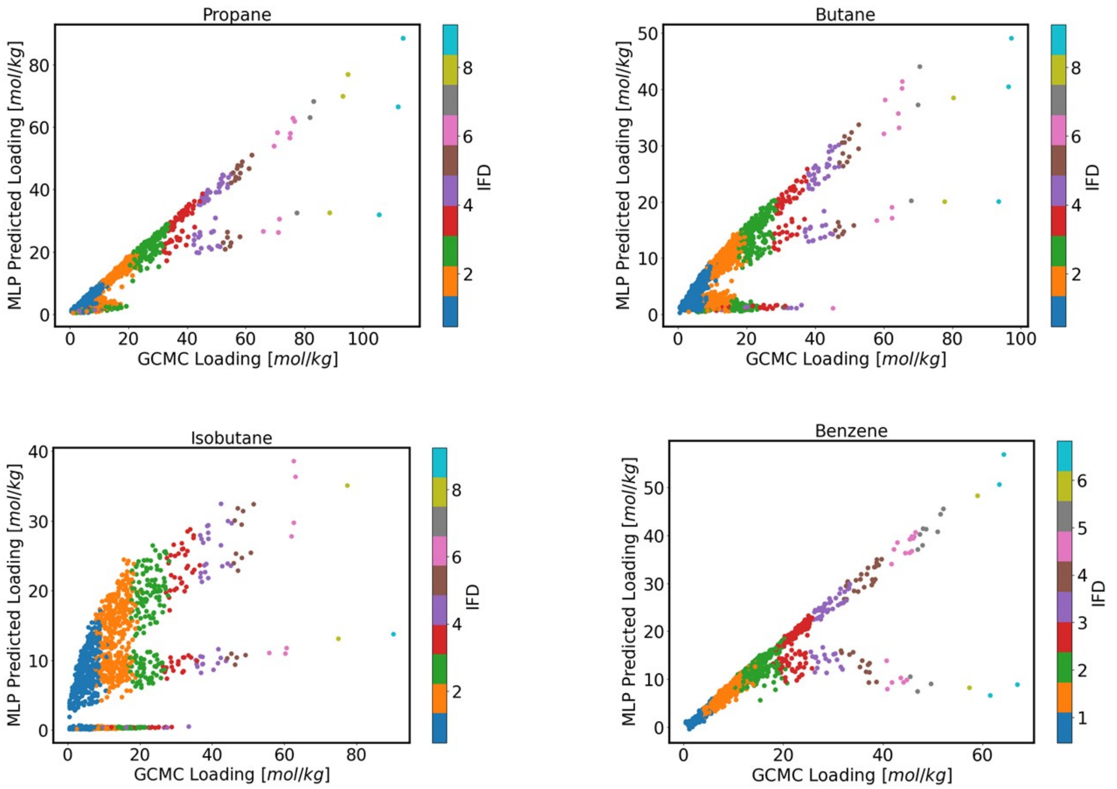

Figure S13. Illustration showcasing the influence of inverse framework density [gcm^{-3}] on the predictive accuracy of adsorption behavior in MOFs.

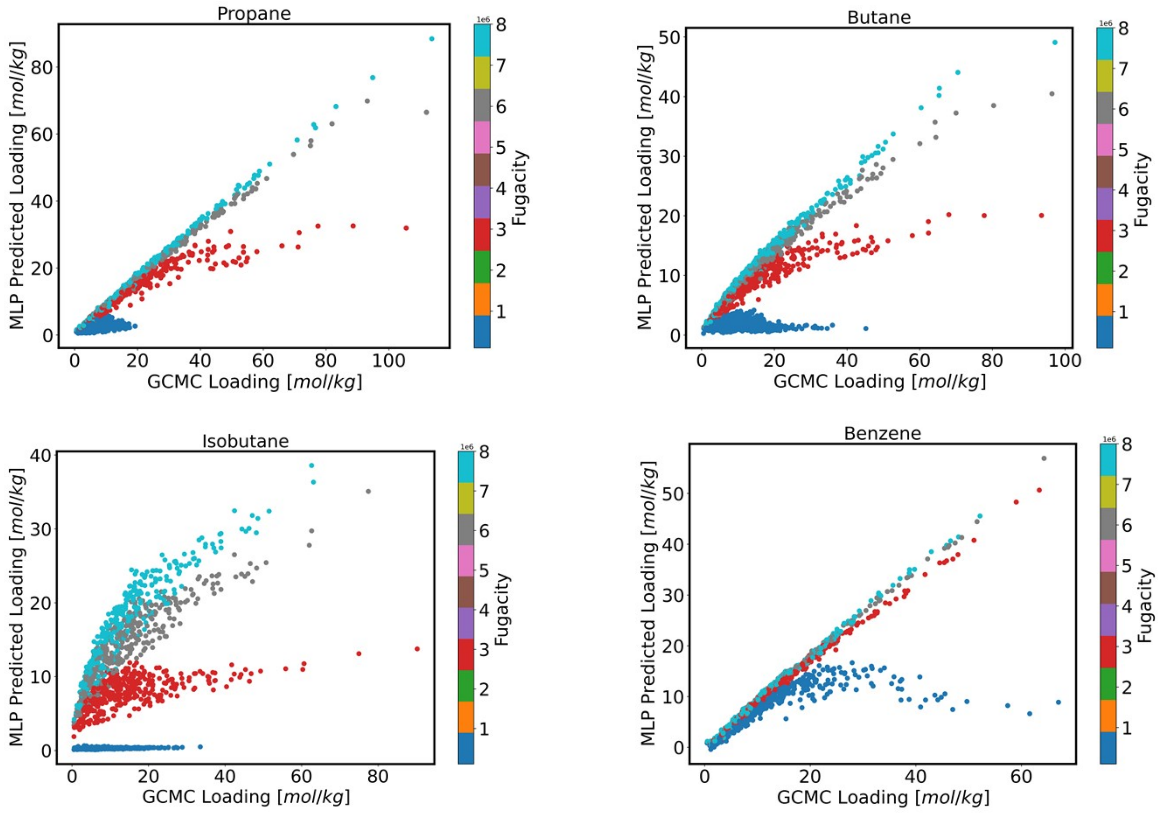

Figure S14. Illustration showcasing the influence of fugacity [Pa] on the predictive accuracy of adsorption behavior in MOFs.

Principal Component Analysis of MOFs

A database of 3445 MOFs and their corresponding textural properties which includes the largest pore diameter, pore limiting diameter, void fraction, surface area, pore size standard deviation and the inverse framework density were converted to two principal components which represented around 91.3% variance. The Principal Component (PC) space and the cumulative explained variance are shown below.

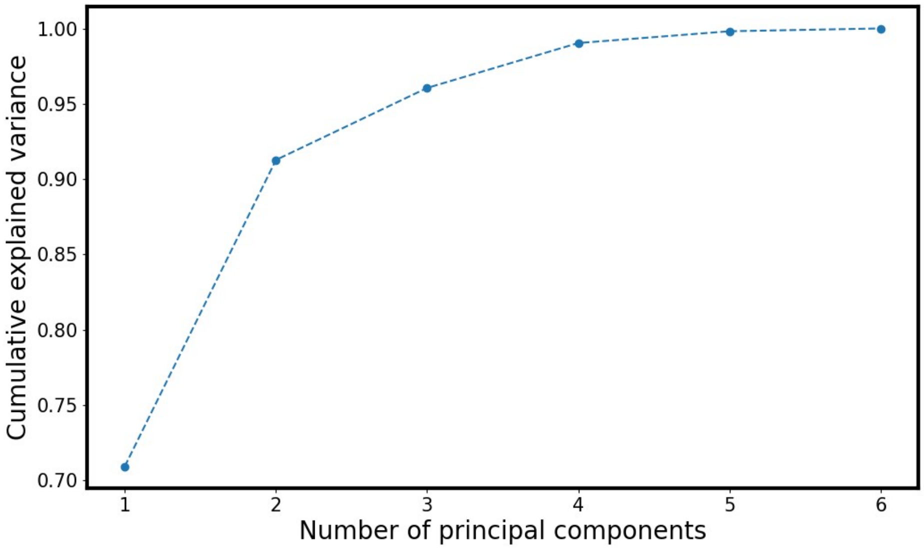

Figure S15. Cumulative explained variance of the principal components generated from the textural properties. 91.3% of the variance was achieved with the first two principal components.

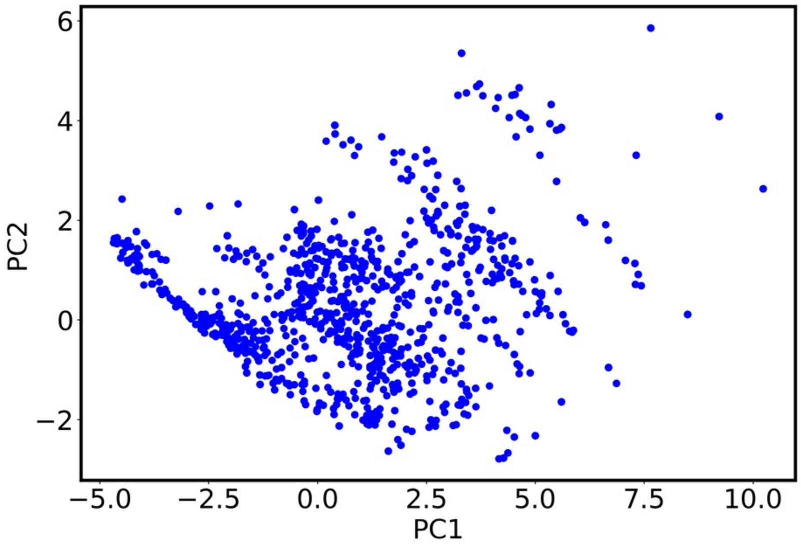

Figure S16. PC1 and PC2 representation of 3445 MOFs.

Active Learning Procedure for simultaneous navigation of fugacity, alchemical, and MOF space.

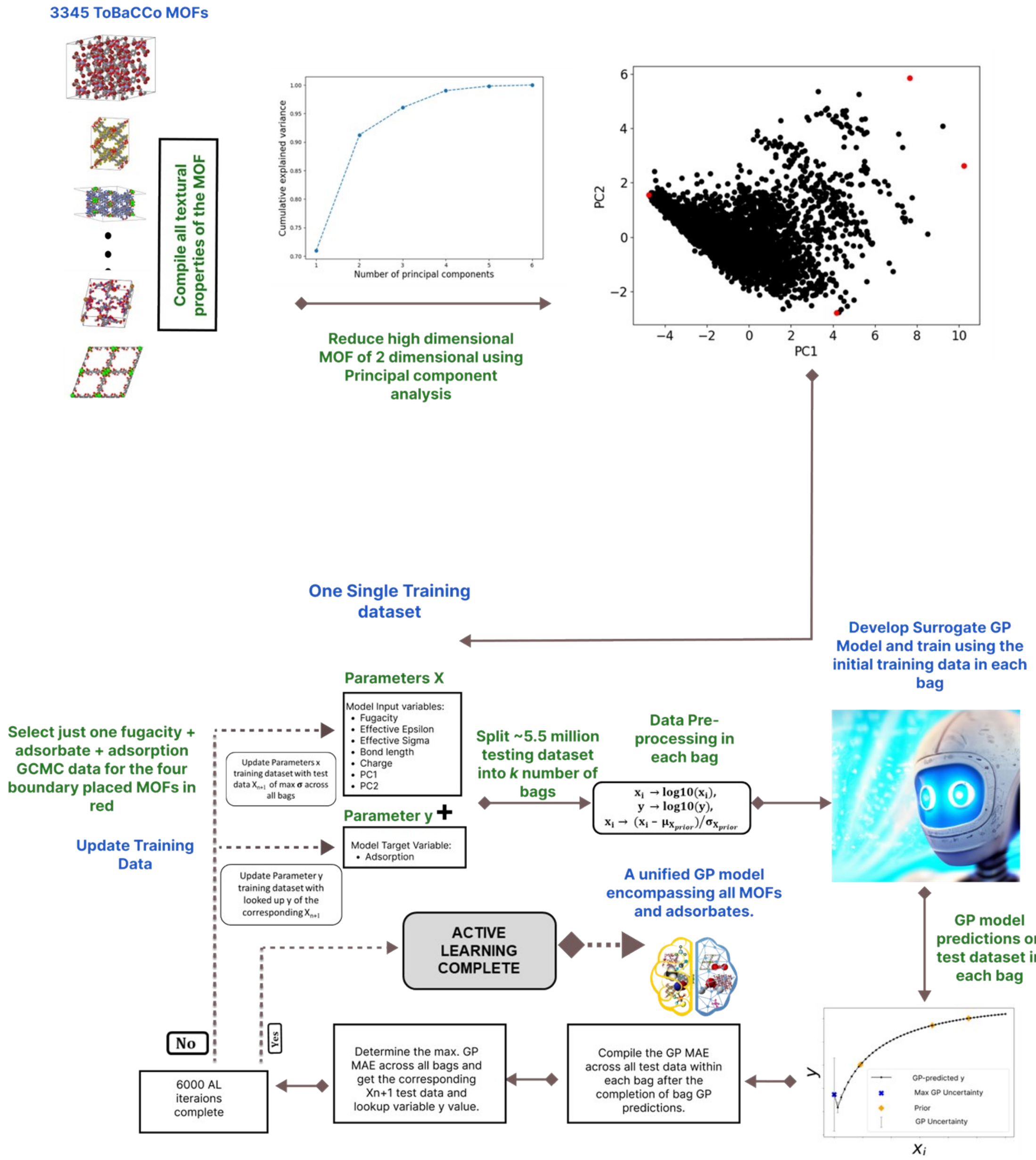

Figure S17. AL framework for MOF and molecule simultaeneous navigation. This framework is used in the simultaeneous navigation of material and adsorbate space with a single GP model. The Bagging approach used in making predictions. Across each bag, the model gets initialized from the starting prior. Points of highest uncertainty across all bags are compiled, and the starting prior is updated with the maximum GP MAE test array across all the bags. This continues till 6000 iterations are completed resulting in a final model of 6004 training data points.

To validate the robustness of the bagging approach, an additional set of 50 bags, each containing a larger volume of data compared to the initial 100 bags used in constructing the GP model, was created. To validate the bagging approach of 100 bags, we created 50 bags in the same fashion and made predictions on the test dataset across these 50 bags which resulted in an impressive average R^2 score of 0.94. This high average R^2 score underscores the accuracy and reliability of the bagging approach, utilizing information from the initial 100 bags. It demonstrates the approach's adeptness in capturing inherent data patterns and relationships, ensuring dependable and consistent predictions.

The single gaussian process principal component analysis (GP-PCA) model was used to make predictions of real molecules within and outside the alchemical training range are shown below.

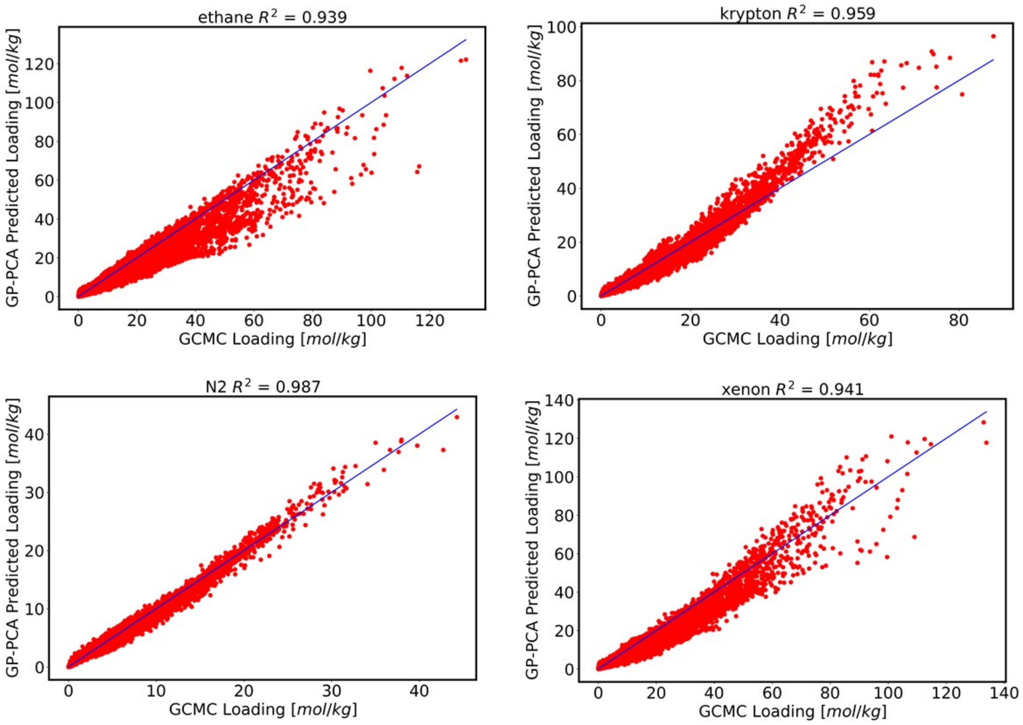

Figure S18. Comparison of GP-PCA model generated from the AL scheme versus GCMC loadings across MOFs. These molecules have their adsorbate features within the alchemical ranges used during the model training.

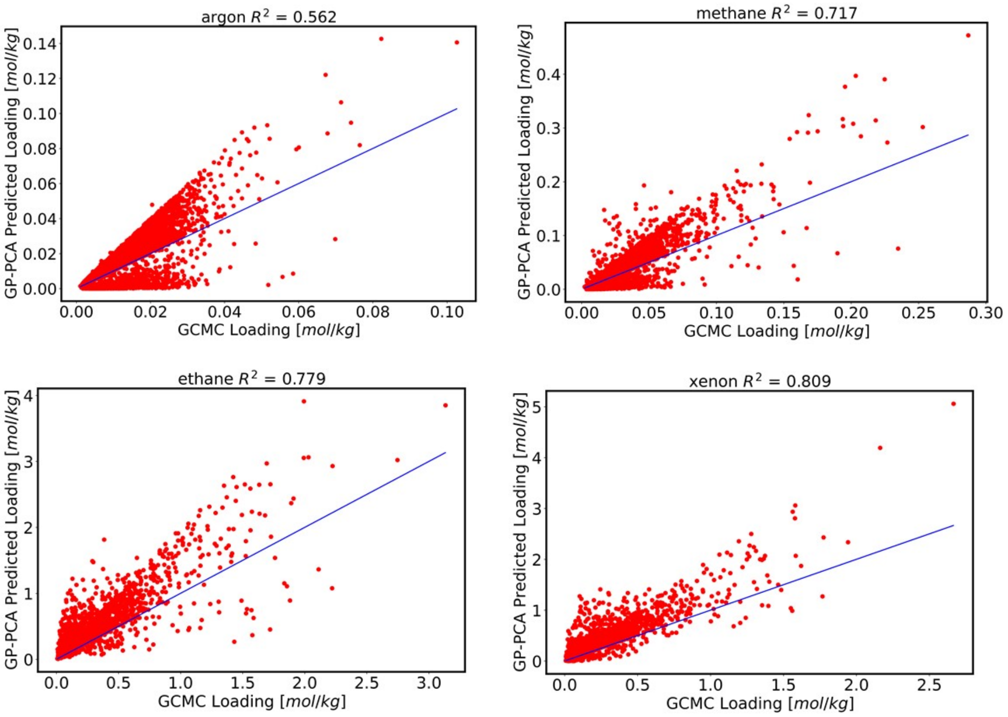

Figure S19. Comparison of GP-PCA model generated from the AL scheme versus GCMC loadings across MOFs, **at low pressures (≤ 0.08 bar).** These molecules have their adsorbate features within the alchemical ranges used during the model training.

Feature Navigation by GP-PCA model

The AL scheme for the GP-PCA model was constructed with 6004 data points consist of the input features: fugacity, effective epsilon, effective sigma, bond length and charges. The plots below show the navigated points using the AL algorithm for the charges, effective epsilon, and the bond length.

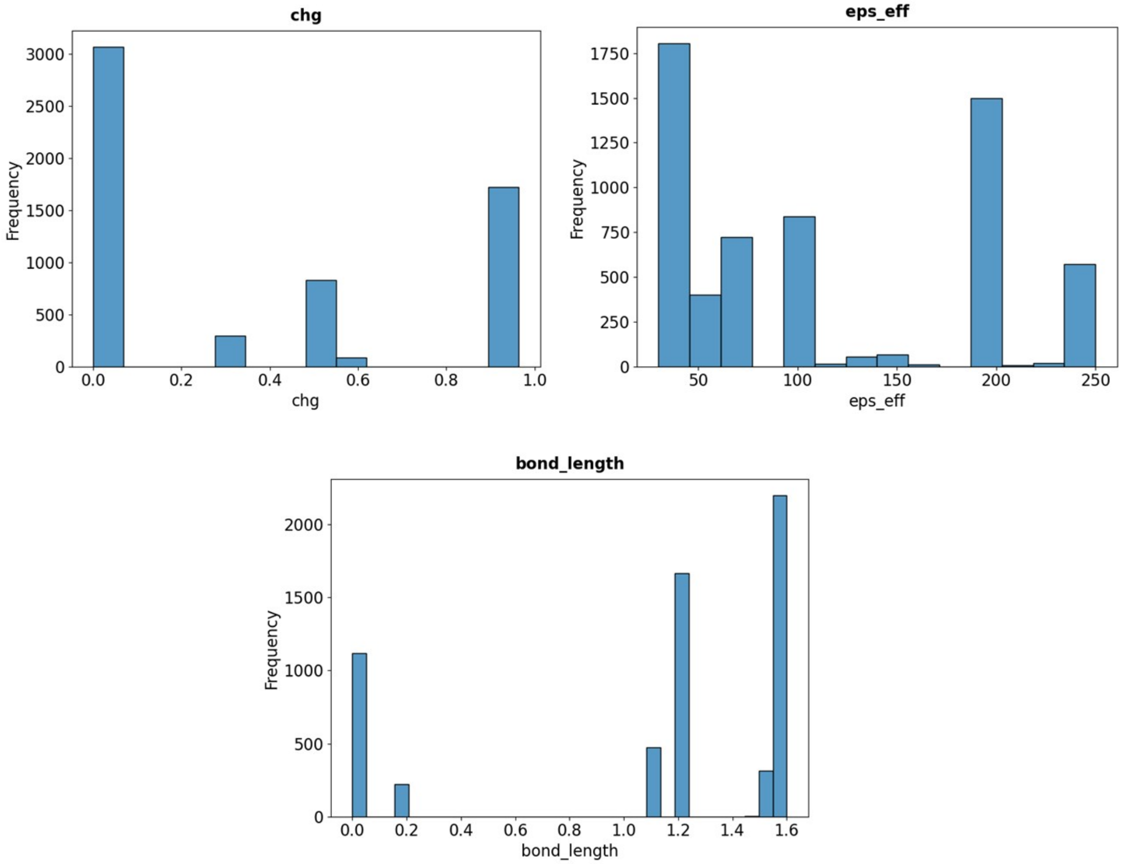

Figure S20. AL sampled regions in the charges, effective epsilon, and bond length.

The AL algorithm also navigated good portions of the textural properties (converted from the PCs) of the MOFs as seen in the figure below.

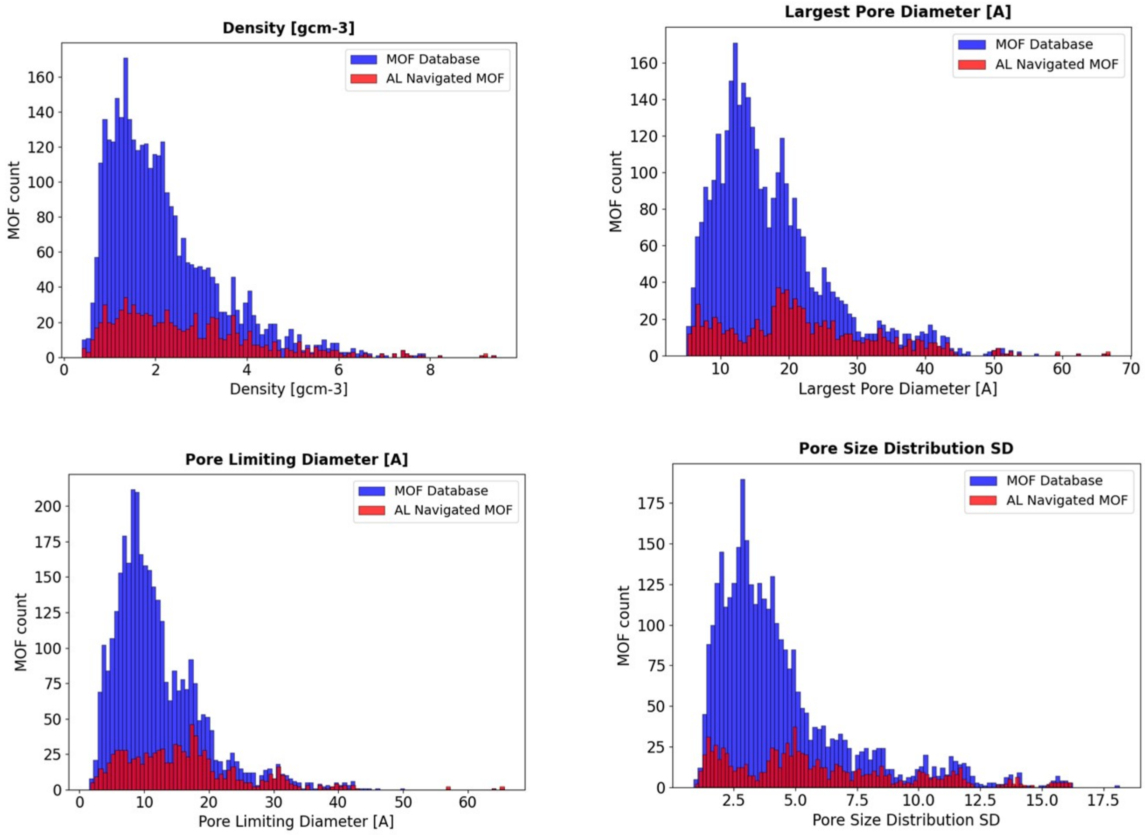

Figure S21. AL sampled regions in terms of textural properties.

MLP model developed from GP-PCA training data.

The final training data from the GP-PCA section was used to build a new MLP model and based on the loss (MAE), the best model was developed with 500 epochs, a batch size of 64 and a learning rate of 0.001. The resulting loss plot (learning curve) is shown below.

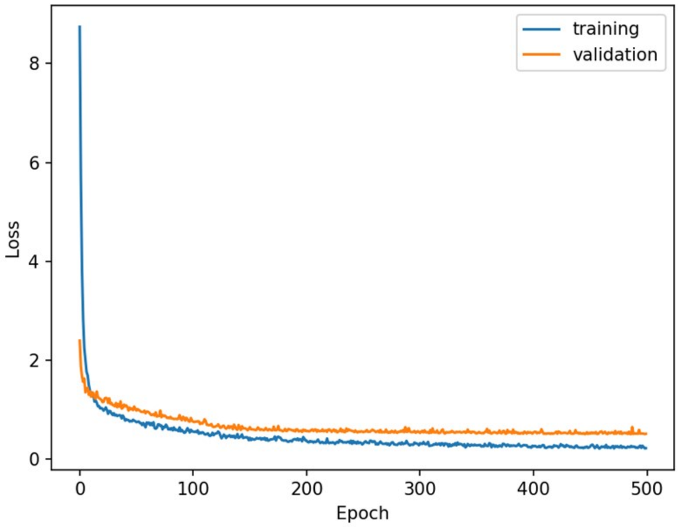

Figure S22: Learning curve for best model from the hyperparameter tuning list.

This model was used to predict the adsorption of real molecules. The results are shown below:

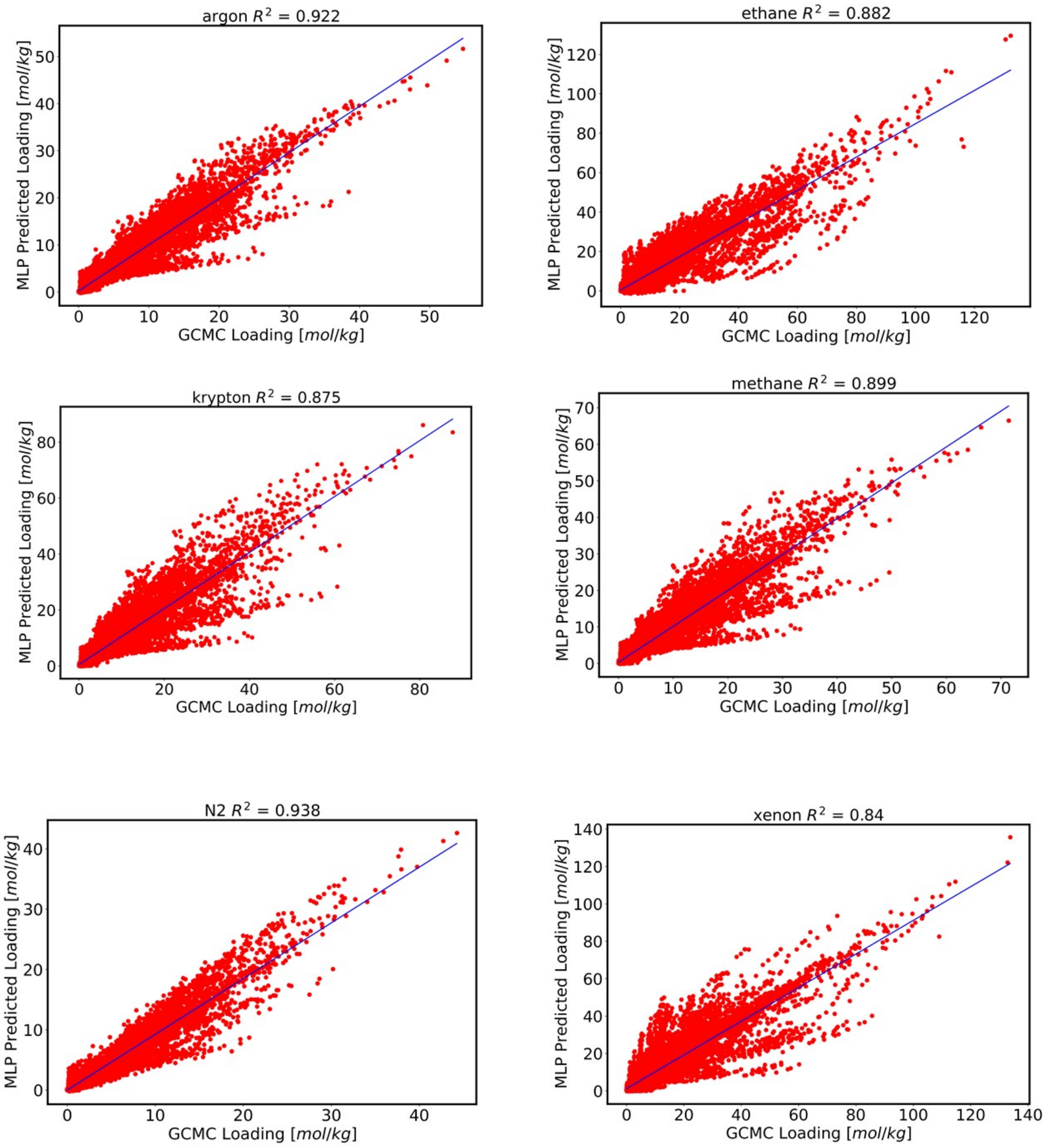

Figure S23. Comparison of MLP model constructed on the GP-PCA training data scheme versus GCMC loadings across MOFs. These molecules have their adsorbate features within the alchemical ranges used during the model training.

Glossary of Machine Learning Jargon.

1. Active Learning (AL) - A subfield of machine learning focused on improving the efficiency of a learning algorithm. AL selectively chooses the most informative data points from which to learn, thereby reducing the number of training data needed and optimizing computational resources.

2. Multilayer Perceptron (MLP) - A type of deep neural network that consists of multiple layers of neurons, each connected with others across layers. MLPs are widely used for classification and regression tasks.

3. Principal Component Analysis (PCA) - A statistical technique used in machine learning to reduce the dimensionality of a data set by transforming it into a new set of variables (principal components) that summarize the key information with a reduced number of dimensions.

4. Gaussian Process Regression (GPR) - A non-parametric kernel-based probabilistic model used in machine learning for regression tasks. GPR predicts not only the continuous output but also the uncertainty of the prediction, making it useful for models where estimating the degree of confidence in the predictions is crucial.

5. Kernel Function- In machine learning, a kernel function is used in various algorithms to enable them to process linearly inseparable data. It implicitly maps input data into higher-dimensional feature spaces.

6. Rational Quadratic (RQ) Kernel - A specific type of kernel used in Gaussian processes, characterized by its ability to model varying scales of variation in the data. It is governed by parameters that control the scale of fluctuations in the data.

7. Surrogate Model- An approximation method that mimics the behavior of a simulation model but at a lower computational cost. Used frequently in scenarios where real-time simulations are computationally expensive or impractical.

8. Bagging - A machine learning ensemble technique designed to improve the stability and accuracy of machine learning algorithms used in statistical classification and regression. It also reduces variance and helps to avoid overfitting.

9. TensorFlow - An open-source software library used for numerical computation and machine learning. TensorFlow offers a flexible and comprehensive ecosystem of tools, libraries, and community resources that let researchers push the state-of-the-art in ML, and developers easily build and deploy ML-powered applications.

10. Training Dataset - A dataset used to train machine learning models. It consists of input data and corresponding target outputs, which the model learns to predict accurately through various learning algorithms.

11. Testing Dataset - A dataset separate from the training data used to evaluate the performance of a trained machine learning model. The testing dataset helps to assess how well a model generalizes to new, unseen data.

12. Epoch - In the context of training neural networks, an epoch refers to one complete pass through the entire training dataset. During an epoch, the model's weights are updated in an effort to minimize the loss function.

13. Batch Size - The number of training examples utilized in one iteration of a model training. Batch size is a crucial parameter in machine learning algorithms, especially in the context of gradient descent, where it balances the speed and stability of the learning process.

14. Learning Rate - A hyperparameter that controls how much to change the model in response to the estimated error each time the model weights are updated. Choosing the right learning rate is crucial for training a model efficiently.

15. Leaky ReLU (Rectified Linear Unit) - A type of activation function used in neural networks, which allows a small, positive gradient when the unit is not active. Leaky ReLU aims to solve the problem of "dying neurons" in neural networks with traditional ReLU activation functions.

16. Ground Truth - The actual, observed value or label in the dataset, used to train machine learning models. In the context of your study, ground truth data would be the real adsorption measurements from experiments or simulations, against which the model predictions are compared.

17. Hyperparameters - Parameters whose values are set before the learning process begins. Hyperparameters, such as learning rate, batch size, and architecture of the model (number of layers, number of neurons per layer), play a crucial role in controlling the behavior of learning algorithms.

18. Model Overfitting - A modeling error in machine learning which occurs when a function is too closely fit to a limited set of data points. Overfitting the training data results in the model learning noise and details that do not generalize to new data.

19. Data Normalization/Standardization - The process of rescaling one or more attributes to have a mean of zero and a standard deviation of one. This is an important pre-processing step for many machine learning algorithms, as it allows the model to train more efficiently.
